# Inhibition of miR‐188‐5p alleviates hepatic fibrosis by significantly reducing the activation and proliferation of HSCs through PTEN/PI3K/AKT pathway

**DOI:** 10.1111/jcmm.16376

**Published:** 2021-03-10

**Authors:** Farooq Riaz, Qian Chen, Kaikai Lu, Ezra Kombo Osoro, Litao Wu, Lina Feng, Rong Zhao, Luyun Yang, Yimeng Zhou, Yingli He, Li Zhu, Xiaojuan Du, Muhammad Sadiq, Xudong Yang, Dongmin Li

**Affiliations:** ^1^ Department of Biochemistry and Molecular Biology School of Basic Medical Sciences Xi’an Jiaotong University Health Science Center Xi’an China; ^2^ Key Laboratory of Environment and Genes Related to Diseases Ministry of Education Xi’an Shaanxi China; ^3^ Department of Infectious Diseases First Affiliated Hospital of Xi’an Jiaotong University Xi’an China

**Keywords:** hepatic fibrosis, hepatic stellate cell, MiR‐188‐5p, non‐alcoholic fatty liver disease, non‐coding RNA, PTEN, AKT pathway

## Abstract

Persistent hepatic damage and chronic inflammation in liver activate the quiescent hepatic stellate cells (HSCs) and cause hepatic fibrosis (HF). Several microRNAs regulate the activation and proliferation of HSCs, thereby playing a critical role in HF progression. Previous studies have reported that miR‐188‐5p is dysregulated during the process of HF. However, the role of miR‐188‐5p in HF remains unclear. This study investigated the potential role of miR‐188‐5p in HSCs and HF. Firstly, we validated the miR‐188‐5p expression in primary cells isolated from liver of carbon tetrachloride (CCl_4_)‐induced mice, TGF‐β1‐induced LX‐2 cells, livers from 6‐month high‐fat diet (HFD)‐induced rat and 4‐month HFD‐induced mice NASH models, and human non‐alcoholic fatty liver disease (NAFLD) patients. Furthermore, we used miR‐188‐5p inhibitors to investigate the therapeutic effects of miR‐188‐5p inhibition in the HFD + CCl_4_ induced in vivo model and the potential role of miR‐188‐5p in the activation and proliferation of HSCs. This present study reported that miR‐188‐5p expression is significantly increased in the human NAFLD, HSCs isolated from liver of CCl_4_ induced mice, and in vitro and in vivo models of HF. Mimicking the miR‐188‐5p resulted in the up‐regulation of HSC activation and proliferation by directly targeting the phosphatase and tensin homolog (PTEN). Moreover, inhibition of miR‐188‐5p reduced the activation and proliferation markers of HSCs through PTEN/AKT pathway. Additionally, in vivo inhibition of miR‐188‐5p suppressed the HF parameters, pro‐fibrotic and pro‐inflammatory genes, and fibrosis. Collectively, our results uncover the pro‐fibrotic role of miR‐188‐5p. Furthermore, we demonstrated that miR‐188‐5p inhibition decreases the severity of HF by reducing the activation and proliferation of HSCs through PTEN/AKT pathway.

## 1 INTRODUCTION

Hepatic fibrosis (HF) and scarring, a serious global health problem, are defined as the common pathological process associated with end‐stage liver cirrhosis and carcinoma.^1^ This process of fibrosis is labelled with persistent hepatic damage, chronic inflammation and wound healing reactions.^2^ During the process of fibrogenesis, activation of several mediators causes the excessive production and agglomeration of extracellular matrix (ECM) components and interstitial collagens lead to HF and scar deposition.^3^, ^4^ Hepatic stellate cells (HSCs) are characterized as the major mesenchymal cells in the liver which play key roles in several cellular processes during HF.^5^ HSCs remain quiescent in healthy individuals; however, following persistent hepatic injury, quiescent HSCs will be activated by trans‐differentiating into fibrogenic myofibroblast‐like cells. This trans‐differentiation promotes the massive accumulation of ECM components, expression of α‐SMA and cell proliferation in response to pro‐inflammatory signals originated from impaired parenchymal cells.^2^, ^4^, ^5^, ^6^ Activated HSCs respond and secrete numerous pro‐fibrogenic cytokines. Among these pro‐fibrotic cytokines, transforming growth factor β (TGF‐β) is considered a potent cytokine resulting in HF.^7^ Thus, inactivation and proliferation inhibition of HSCs have been widely accepted to obstruct the HF.^8^

Non‐coding RNAs (ncRNA), group of evolutionary conserved endogenous RNAs, influence gene expression and may regulate initiation and progression of NASH (non‐alcoholic steatohepatitis) and HF.^9^ Among these regulatory molecules, microRNAs (miRNAs), short ≈ 22 nucleotides in size, negatively regulate expressions of target genes at the post‐transcriptional level by targeting 3′ untranslated regions (3′ UTR) of their target mRNAs.^10^, ^11^ Several studies have explored the fundamental roles of miRNAs in the progression of HF by regulating several cellular processes including HSC activation, proliferation and collagen production.^9^, ^12^ For example, overexpression of miR‐193a/b‐3p inhibited TGF‐β1‐induced activation and proliferation of HSCs by suppressing HSC activation genes COL1A1 and α‐SMA, and attenuated HF.^13^ Likewise, miR‐146a‐5p inhibited the secretion of pro‐inflammatory factors and activation of HSCs suggesting the therapeutic role of miR‐146a‐5p in HF.^14^ Hyun et al investigated that the expression of miR‐188‐5p is significantly up‐regulated in carbon tetrachloride (CCl_4_)‐induced HF model.^15^, ^16^ However, the functional importance of miR‐188‐5p in HF is still unclear. This study aimed to determine the expression of miR‐188‐5p in the TGF‐β1‐induced HSCs and the fibrotic livers, and investigated the role of miR‐188‐5p in modulating the activation, proliferation and fibrogenesis of HSCs, both in vitro and in vivo.

## 2 MATERIAL AND METHODS

### 2.1 Human liver biopsy samples

Human liver biopsy samples were obtained from five patients with chronic hepatitis B (CHB) and non‐alcoholic fatty liver disease (NAFLD) and four CHB patients in the First Affiliated Hospital of Xi'an Jiaotong University. Written informed consents were signed from each patient before obtaining liver biopsy samples. Diagnosis was made on the basis of typical morphological findings using ultrasonography. The study involved human samples was approved by the Clinical Research Ethics Committee of The First Affiliated Hospital of Xi'an Jiaotong University and adhered to the principles outlined in the Declaration of Helsinki. The clinical characteristics of subjects included in this study are listed in Table 1.

**TABLE 1:** Clinical characteristics of Human subjects used for this study

Factors	CHB	CHB + NAFLD
N	4	5
Male, n (%)	3 (75)	5 (100)
Mean age in years at biopsy	35.5 (4.31)	30 (5.94)
Laboratory measures, mean (SD)		
Serum AST (IU/L)	19 (0.63)	29 (100.83)
Serum ALT (IU/L)	18 (3.03)	64 (40.56)
Serum ALP (IU/L)	59 (7.73)	79 (182.75)
Total bilirubin (μmol/L)	13.9 (3.09)	12.4 (78.36)
Direct bilirubin (μmol/L)	3.85 (0.85)	4.9 (60.54)
Serum GGT (IU/L)	9.5 (2.25)	18 (2.014)

Note

Values represent as mean ± SEM.

### 2.2 Animals and liver specimens

Adult male C57BL/6J mice and E3 rats were obtained from the Experimental Animal Center situated in Xi'an Jiaotong University. All the animals were housed in a specific pathogen‐free animal facility centre with controlled temperature and humidity, and maintained under 12‐h light/dark cycle with free access to water and feed. All animal experimental protocols were carried out in accordance with the European Communities Council Directive 2010/63/EU for the protection of animals used for scientific purposes and approved by the Institutional Ethics Committee in School of Medicine of Xi'an Jiaotong University (No. XJ2013086).

To determine the expression of miR‐188‐5p in high‐fat diet (HFD)‐induced liver fibrosis models, livers were obtained from 8 control and 8 4‐month HFD‐induced C57BL/6J mice (Research Diet, D12492), and 8 control and 8 6‐month HFD‐induced E3 rats (described in our previous experiments^17^).

### 2.3 Isolation of primary cells

We used 8‐week‐old male C57BL/6J mice to isolate primary cells. Briefly, we randomly divided mice into control group and CCl_4_ (2 mL/kg body weight)‐induced chronic liver injury model group (n = 6 each group). As described earlier,^18^ hepatocytes and non‐parenchymal cells (NPC) were isolated using differential centrifugation to evaluate the expression of miR‐188‐5p.

### 2.4 Inhibition of miR‐188‐5p in vivo

To investigate the effect of miR‐188‐5p inhibition on HF in vivo, we used diet and chemical‐induced dual fibrosis model. In several studies, mild dose of intraperitoneal (i.p.) CCl_4_ injections to animals fed on HFD has been accepted as a promised and rapid liver fibrosis model by triggering inflammation, steatosis and fibrosis which are histological features of NASH.^19^, ^20^ Therefore, using the same strategy, 40 male C57BL/6J mice were randomized into five groups (n = 8 mice in each group): Control group, mice were fed on normal diet; HFD group, mice were fed on HFD (Research Diet, D12492) along with weekly 200 μl i.p. injection of corn oil (Shanghai Aladdin Biochemical, C116025) for 14 weeks; HFD + CCl_4_ group, mice were fed with HFD along with weekly i.p. administration of CCL_4_ (Tianli Chemical Reagent, GB/T688‐2011) diluted in corn oil (CCl_4_ at dose rate of 0.25 μl (0.40 μg)/g of body weight diluted in 200 μl of corn oil) for 14 weeks; HFD + CCl_4_‐miR‐NC group; and HFD + CCl_4_‐miR‐188‐5p‐inhibitor group. To observe the anti‐fibrotic effect of miR‐188‐5p inhibition, 62.5 nM/kg dose of miR‐188 inhibitors or miR‐NC complexed with Lipofectamine^®^ 3000 (Invitrogen, L3000015) were diluted in 50 μl of sterile normal saline and injected in respective groups of mice through tail vein injection at the mid of 5^th^ week, start of 6^th^ and 7^th^ week. The schematic representation of animal model groups used in the study for the inhibition of miR‐188‐5p is presented in Figure S2. At the 14^th^ week of experiment, all mice were sacrificed. Liver tissues were collected and divided into two parts. One part was immediately snap‐frozen in liquid nitrogen and then stored at −80°C for further experiments, while another section was fixed in formalin and embedded in paraffin, for histological analysis.

### 2.5 Statistical analysis

Quantitative data were presented as means ± SEM. Statistical analysis between different groups was assessed by GraphPad Prism 6.02 (GraphPad Software) Software. Student's t test and two‐way ANOVA with Tukey's multiple comparison tests were performed to compare two groups or multiple groups, respectively.

Detailed materials and methods are described in Supporting information.

## 3 RESULTS

### 3.1 Expression of miR‐188‐5p is up‐regulated in human liver fibrosis biopsy sample and in vivo and in vitro models for liver fibrosis

**FIGURE 1:**
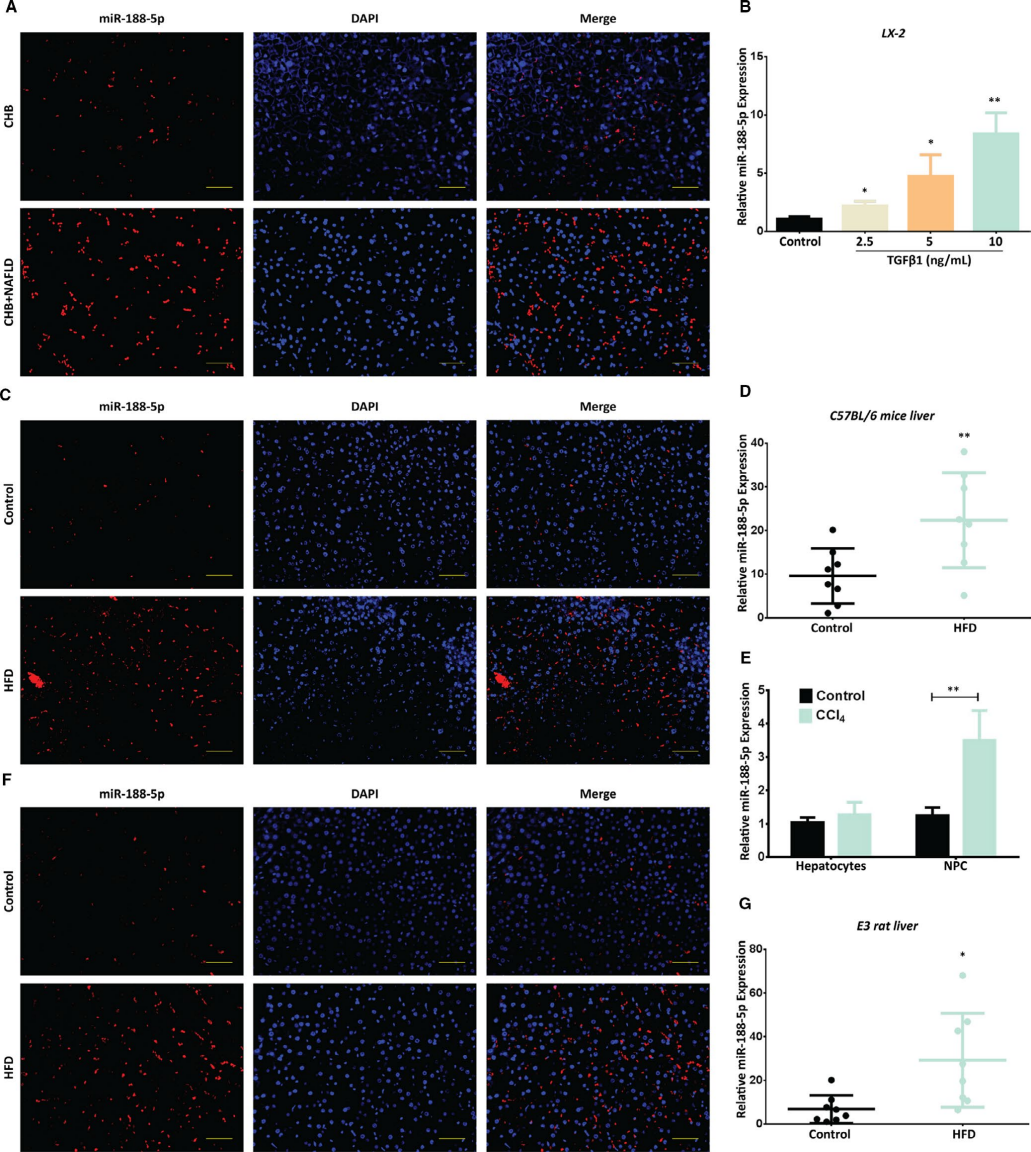
Increased expression of miR‐188‐5p in in vitro and in vivo models of hepatic fibrosis. A, Representative FISH images showing the expression of miR‐188‐5p in human livers from chronic hepatitis B (CHB) patients (n = 4) and CHB + NAFLD patients (n = 5). B, Differential expression of miR‐188‐5p in TGFβ‐1‐induced LX‐2 cells was validated using RT‐qPCR. C and D, Representative FISH images (C) and RT‐qPCR analysis (D) showing the expression of miR‐188‐5p in the liver specimen of 4‐month HFD‐induced C57BL/6 mice (n = 8). E, Differential expression of miR‐188‐5p in primary hepatocytes and non‐parenchymal cells (NPCs) isolated from livers of CCl_4_‐induced mice and control mice was validated using RT‐qPCR. F and G, Representative FISH images (F) and RT‐qPCR analysis (G) showing the expression of miR‐188‐5p in the liver specimen of 6‐month HFD‐induced E3 rats (n = 8). Values represent as mean ± SEM, *P  < 0.05 and **P  < 0.01

Several investigations have reported that miR‐188‐5p plays critical role in numerous diseases.^21^, ^22^, ^23^, ^24^, ^25^, ^26^, ^27^, ^28^ Recent miRNA signature showed that miR‐188‐5p is up‐regulated in the serum from alcohol‐induced liver cirrhosis and toxic doses of acetaminophen (APAP) liver injury patients. However, the expression of miR‐188‐5p was down‐regulated in the type 2 diabetes mellitus patients, while no change was observed in the expression of miR‐188‐5p from HBV patients.^29^ Moreover, it has been identified that miR‐188‐5p is significantly up‐regulated in the HSCs associated with portal hypertension and CCl_4_‐induced liver fibrosis model.^15^, ^30^ However, it is still unclear how miR‐188‐5p takes part in the progression of liver fibrosis. In order to explore the role of miR‐188‐5p in liver fibrosis, we first determined the relative expression of miR‐188‐5p in human liver fibrosis biopsy sample from 5 CHB + NAFLD and 4 CHB patients and in vivo and in vitro models for liver fibrosis. We used miR‐188‐5p‐fluorescence in‐situ hybridization (FISH) to determine the expression of miR‐188‐5p in the human HF tissue specimens. We observed that the miR‐188‐5p expression was up‐regulated in the human liver biopsy sample from CHB + NAFLD patients (Figure 1A). To further determine the expression of miR‐188‐5p in human HSCs, we induced LX‐2 cells with TGF‐β1, which is one of the most important inflammatory mediator and activator of HSCs.^31^ We observed that the expression levels of α‐SMA, Col1α2 and Col3α1 were increased in LX‐2 cells induced with different doses of TGF‐β1 (0, 2.5, 5, 10 ng/mL) at transcript level (Figure S1A‐C). Moreover, the protein expression levels of α‐SMA and Col1α2 were also increased in LX‐2 cells induced with similar doses of TGF‐β1 at protein level (Figure S1D‐F). This suggested that TGF‐β1‐induced LX‐2 cells were associated with the up‐regulation of α‐SMA, Col1 and Col3, which are among the important characteristics and markers for HSC activation and liver fibrosis, and TGF‐β1 successfully induced the activation of HSC cells in vitro. Then we evaluated the expression of miR‐188‐5p in LX‐2 cells induced with different doses of TGF‐β1 by RT‐quantitative polymerase chain reaction (RT‐qPCR). It showed a dose‐dependent increase in the expression of miR‐188‐5p in response to TGF‐β1 (Figure 1B), the highest expression level, especially at 10 ng/mL of TGF‐β1. To explore the role of miR‐188‐5p in liver fibrosis, LX‐2 cells were treated with 10 ng/mL of TGF‐β1 for further experiments. In addition, miR‐188‐5p expression was determined in the liver specimens of 4‐month HFD‐induced C57BL/6J mice and 6‐month HFD‐induced E3 rats. FISH images show increase in the expression of miR‐188‐5 in the livers of 4‐month HFD‐induced C57BL/6J mice (Figure 1C) and 6‐month HFD‐induced E3 rats (Figure 1F). Similarly, the results obtained from RT‐qPCR analysis showed that miR‐188‐5p expression was significantly up‐regulated among HFD groups as compared to their respective control groups (Figure 1D,G). Furthermore, RT‐qPCR analysis showed that miR‐188‐5p expression was significantly up‐regulated in the NPC cells isolated from liver of CCl_4_‐induced mice as compared to control, whereas no significant change was observed in primary hepatocytes isolated from liver of CCl_4_‐induced mice (Figure 1E). These data indicated that miR‐188‐5p is highly expressed in activated HSCs and in vivo models of liver fibrosis.

### 3.2 Up‐regulation of miR‐188‐5p promotes HSC activation and proliferation in LX2 cells

**FIGURE 2:**
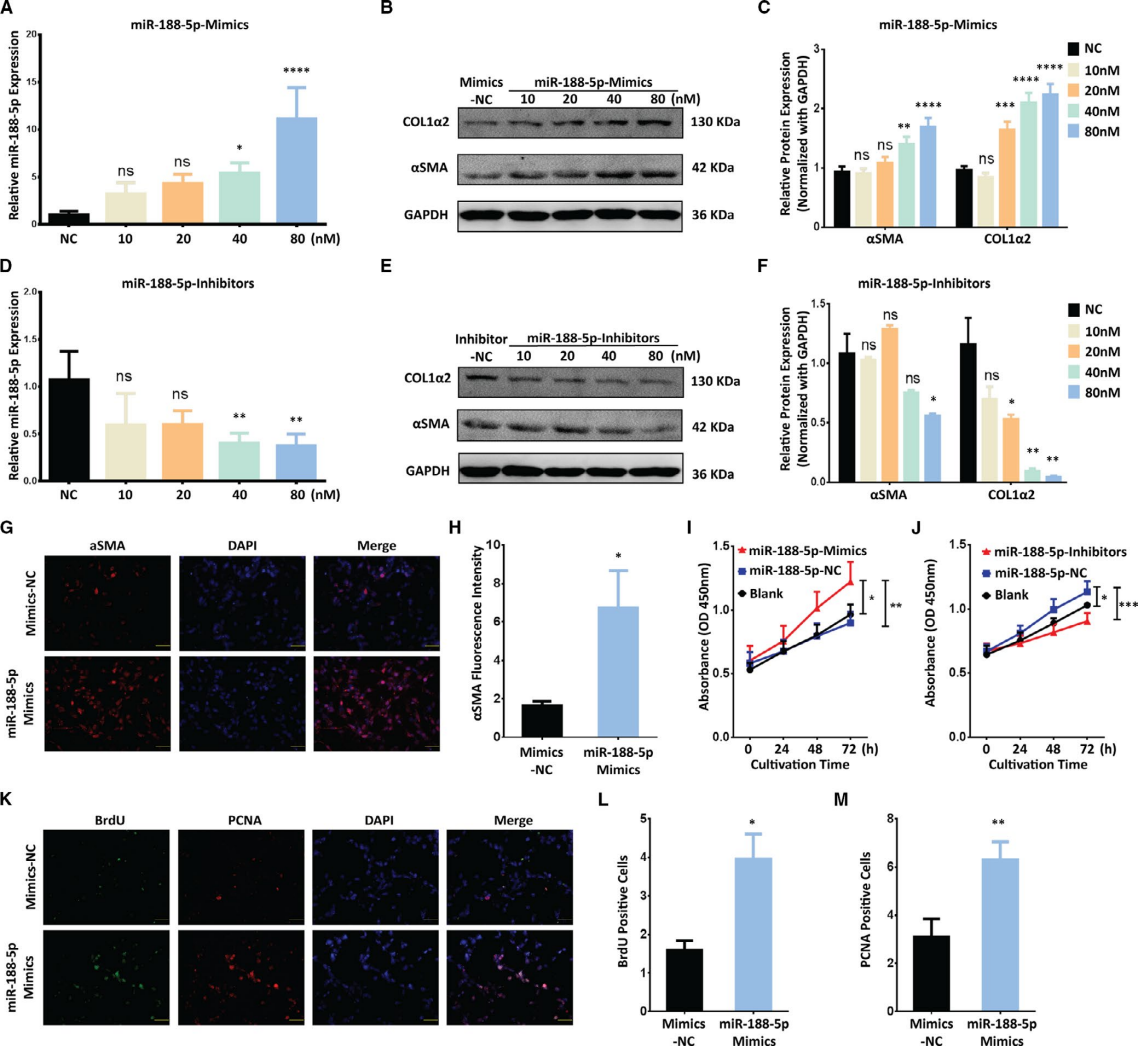
MiR‐188‐5p promotes the activation and proliferation of LX‐2 cells. A, RT‐qPCR analysis of miR‐188‐5p in LX‐2 cells transfected with different doses of miR‐188‐5p mimics. B and C, Western blotting analysis (B) and quantification analysis (C) of the protein expression of HSC activation marker and pro‐fibrotic gene α‐SMA and COL1A2 in LX‐2 cells transfected with different doses of miR‐188‐5p‐mimics. D, RT‐qPCR analysis of miR‐188‐5p in LX‐2cells transfected with different doses of miR‐188‐5p‐inhibitors showing relative expression of miR‐188‐5p. E and F, Western blotting analysis (E) and quantification analysis (F) showing the protein expression of HSC activation marker and pro‐fibrotic gene α‐SMA and COL1A2 in LX‐2 cells transfected with different doses of miR‐188‐5p‐inhibitors. G, Representative immunofluorescence images showing the expression of HSC activation marker and pro‐fibrotic gene α‐SMA (scale bar 50 µm) (G) and the quantification of α‐SMA fluorescence intensity (H) in LX‐2 cells transfected with miR‐188‐5p‐mimics. I and J, The cell proliferation activity of LX‐2 cells detected using CCK‐8 kit after transfection with miR‐188‐5p‐mimics (I) or miR‐188‐5p‐inhibitors (J). K, BrdU incorporation assay showing the dual‐positive (scale bar 50 µm) LX‐2 cells after transfection of miR‐188‐5p‐mimics. L and M, Quantification of BrdU (L) and PCNA (M) positive LX‐2 cellstransfected with miR‐188‐5p‐mimics. Values represent as mean ± SEM. *P < 0.05, **P < 0.01, ***P < 0.001 and ****P < 0.0001

To explore the functional relevance of miR‐188‐5p in liver fibrosis, we modulated the expression of miR‐188‐5p using mimics or inhibitors in LX‐2 cells. Firstly, the overexpression of miR‐188‐5p via mimics up‐regulated the expression level of miR‐188‐5p (Figure 2A). This overexpression of miR‐188‐5p promoted the expression of HSC activation markers α‐SMA and Col1α2 at protein level (Figure 2B,C). On the contrary, miR‐188‐5p expression was significantly decreased in LX‐2 cells transfected with miR‐188‐5p inhibitors, compared to the negative control (NC; Figure 2D). Furthermore, the protein level of α‐SMA and Col1α2 was significantly reduced following the inhibition of miR‐188‐5p in LX‐2 cells (Figure 2E,F). To support our data, we further evaluated the expression of α‐SMA in LX‐2 cells treated with miR‐188‐5p mimics through the immunofluorescence (IF). Coherent with our finding, miR‐188‐5p mimics significantly up‐regulated the HSC activation marker α‐SMA (Figure 2G,H). These results validated the functional relevance of miR‐188‐5p in activation of HSCs and liver fibrosis in vitro. Furthermore, CCK‐8 assay showed that overexpression of miR‐188‐5p could significantly promote cell proliferation in LX2 cells (Figure 2I). In contrast, cell proliferation activity was significantly suppressed in human LX‐2 cells after the inhibition of miR‐188‐5p compared with that in control group (Figure 2J). To further validate the role of miR‐188‐5p in cell proliferation, we used bromodeoxyuridine (BrdU) dual IF assay using BrdU and proliferating cell nuclear antigen (PCNA)‐specific antibodies. It has been shown that miR‐188‐5p mimics significantly increased the BrdU and PCNA positive cells compared to the control (Figure 2K‐M). It suggested that miR‐188‐5p plays a critical role in regulating the proliferation of HSCs. Overall, these results indicate that up‐regulation of miR‐188‐5p promotes the activation and proliferation of HSCs.

### 3.3 Modulation in miR‐188‐5p expression regulates the PTEN‐dependent proliferation and activation of LX‐2 cells by targeting PI3K/AKT pathway

**FIGURE 3:**
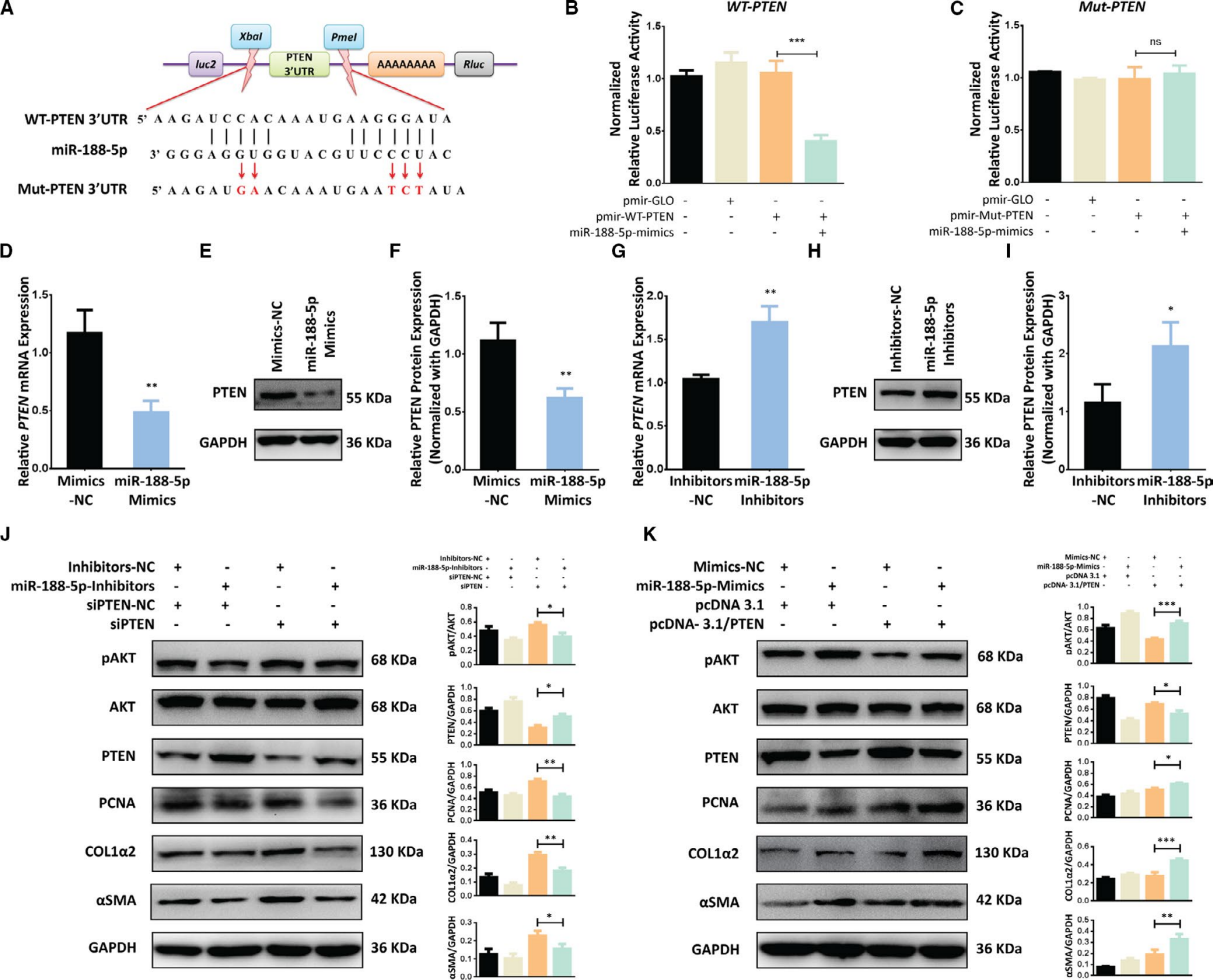
MiR‐188‐5p regulates the activation and proliferation of LX‐2 cells through PTEN/AKT pathway by directly targeting the 3′UTR of PTEN. A, Schematic representation of pmiR‐GLO luciferase reporter constructs harbouring PTEN 3′UTR targeted by miR‐188‐5p and the mutated bases of Mut‐PTEN 3′UTR. B, Relative luciferase activity unit (RLU) showing the luciferase activity of pmir‐WT‐PTEN3′UTR co‐transfected with miR‐188‐5p mimics. C, Relative luciferase activity (RLU) showing the luciferase activity of pmir‐Mut‐PTEN3′UTR co‐transfected with miR‐188‐5p mimics. D, mRNA expression of PTEN in LX‐2 cells transfected with miR‐188‐5p mimics. E. Western blotting analysis showing the PTEN expression in LX‐2 cells transfected with miR‐188‐5p‐mimics. F. Quantified PTEN expression in LX‐2 cells transfected with miR‐188‐5p‐mimics. G. mRNA expression of PTEN in LX‐2 cells transfected with miR‐188‐5p‐inhibitors. H. Western blot analysis showing the PTEN expression in the LX‐2 cells transfected with miR‐188‐5p‐inhibitors. I. Quantified PTEN expression in the LX‐2 cells transfected with miR‐188‐5p‐inhibitors. J, Western blotting analysis showing the effects of miR‐188‐5p‐inhibitors on expression of HSC activation marker α‐SMA and COL1A2, proliferation marker PCNA and PTEN/pAKT pathway in LX‐2 cells when co‐transfected with siPTEN. K, Western blotting analysis showing the effects of miR‐188‐5p‐mimics on expression of HSC activation marker α‐SMA and COL1A2, proliferation marker PCNA and PTEN/pAKT pathway in LX‐2 cells when co‐transfected with pCNA‐3.1/PTEN overexpression plasmid. Values represent as mean ± SEM. *P < 0.05, **P < 0.01and ***P < 0.001

It is known that miRNAs regulate protein expressions through mRNA cleavage or translational repression by pairing with the 3′‐UTR of their respective target genes.^10^ Among the genes common to all databases for miR target prediction, we chose PTEN, a key tumour suppressor, as a target for further research because of its vital role in cell proliferation.^32^ PTEN might be a potential target of miR‐188‐5p, which contained a miR‐188‐5p binding site in 3′UTR region. Homology examination showed that the predicted 8 mer site in the seed region of miR‐188‐5p were complementary to bases of PTEN (Figure 3A). No perfect binding site was predicted or identified for miR‐188‐5p in the coding region of PTEN. To determine whether miR‐188‐5p directly binds to the predicated site of the PTEN, we performed luciferase assay using either the wild‐type 3′‐UTR cloned in pmir‐GLO vector (pmir‐WT‐PTEN‐3’UTR) or the mutant 3′‐UTR (pmir‐Mut‐PTEN‐3′UTR) lacking the miR‐188‐5p binding site (Figure 3A). MiR‐188‐5p mimics significantly reduced PTEN 3′UTR‐dependent luciferase activity (Figure 3B), but it did not affect the pmir‐PTEN‐Mutant‐dependent luciferase activity (Figure 3C). These results suggested there is a direct binding between miR‐188‐5p and the 3’‐UTR of PTEN mRNA. To further confirm either, miR‐188‐5p directly influences the expression of PTEN, we used Western blotting and RT‐qPCR analysis, and found that PTEN expression was significantly decreased in LX‐2 cells transfected with miR‐188‐5p mimics at protein and mRNA level (Figure 3D‐F). In contrast, PTEN expression was significantly increased in LX‐2 cells transfected with miR‐188‐5p inhibitors at protein and mRNA level (Figure 3G‐I). These results indicate that PTEN is a direct target of miR‐188‐5p.

The above findings brought us that miR‐188‐5p might regulate the activation and proliferation of HSC by targeting PTEN. To verify the potential role of miR‐188‐5p in PTEN‐dependent regulation of HSC activation and proliferation, we used co‐transfection of siPTEN and miR‐188‐5p inhibitors. The silencing of PTEN expression, through siPTEN, resulted in the down‐regulation of PTEN, and up‐regulation of HSC activation markers α‐SMA and Col1α2 and HSC proliferation marker PCNA (Figure 3J). On the contrary, miR188‐p inhibitors transfected along with siPTEN reversed the activity of siPTEN and underwent the down‐regulation of α‐SMA, Col1α2 and PCNA (Figure 3J). To further verify our finding, we used PTEN overexpression plasmid to overexpress PTEN in LX‐2 cells. We found that overexpression of PTEN resulted in up‐regulation of PTEN and down‐regulation of HSC activation marker α‐SMA and Col1α2 and proliferation marker PCNA in human LX‐2 cells. However, LX‐2 cells co‐transfected with PTEN overexpression plasmid and miR‐188‐5p mimics significantly down‐regulated the expression of PTEN as compared to cells transfected with PTEN overexpression plasmid alone (Figure 3K). Conversely, the expression of HSC activation markers α‐SMA and Col1α2 was significantly up‐regulated in the cells co‐transfected with pcDNA‐3.1/PTEN overexpression plasmid and miR‐188‐5p mimics (Figure 3K). Furthermore, LX‐2 cell proliferation marker PCNA was also reduced by overexpression of PTEN, which was subsequently improved by co‐transfection of miR‐188‐5p mimics and PTEN overexpression plasmid (Figure 3K).

In addition, siPTEN induces the phosphorylation of AKT, but co‐transfection of siPTEN with miR‐188‐5p inhibitors reduced the expression of pAKT (Phosphorylated AKT; Figure 3J). Likewise, PTEN overexpression reduces the expression of pAKT; however, co‐transfection of PTEN OE plasmid with miR‐188‐5p mimics induced the expression of pAKT (Figure 3K). Collectively our results show that miR‐188‐5p modulates the expression of PTEN in HSCs and regulate the PTEN‐dependent activation and proliferation of HSCs through PI3K/AKT pathway.

### 3.4 Inhibition of miR‐188‐5p reduces the severity of hepatic damage in HFD + CCl_4_‐induced liver fibrosis mice

**FIGURE 4:**
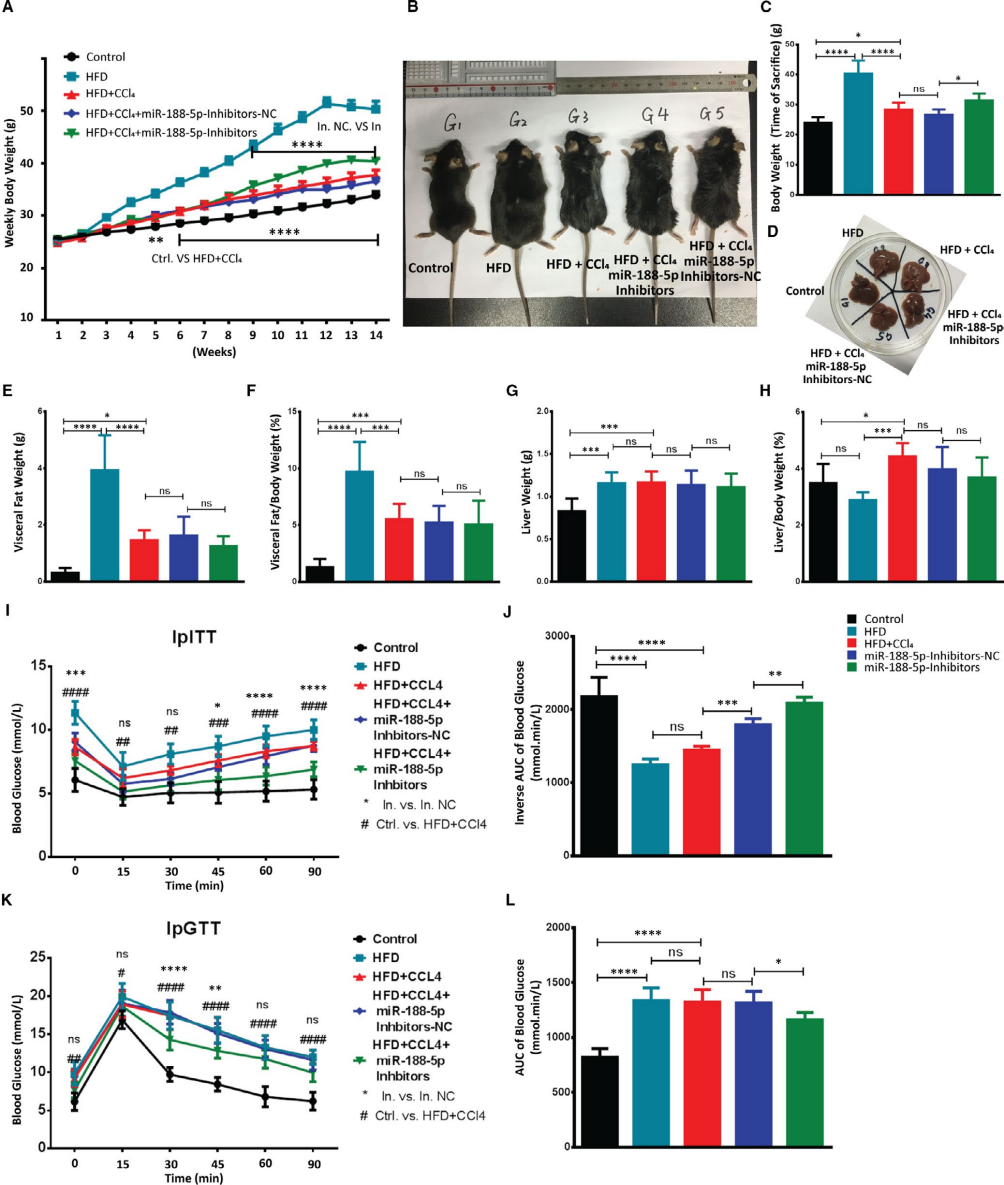
Inhibition of miR‐188‐5p restores the metabolic phenotyping characteristics in HFD + CCl4‐induced hepatic fibrosis. A, Weekly weight gain (g) in different animal groups used for in vivo studies. B–D, Representative image of mice (B), bar graph showing the weight (g) of mice (C) and representative image of mice liver (D) from each group at the time of sacrifice. E‐H, Visceral fat weight (g) (E), visceral fat/body weight (%) (F), liver weight (g) (G) and liver/body weight (%) (H) showed in different animal groups used for in vivo studies. I, Glucose level after the administration of i.p. insulin. J, Inverse area under the curve (AUC) of ipITT showing the insulin resistance. K, Glucose level after the administration of i.p. glucose. L, AUC of ipGTT showing the glucose tolerance in different animal groups used for in vivo studies (n = 8 each group). Values represent as mean ± SEM. *, # represents *P* < 0.05 and **, ## represents *P*  < 0.01, ***, ### represents *P*  < 0.001, and ****, #### represents *P*  < 0.0001

To determine the biological roles of miR‐188‐5p in liver fibrosis, we subjected C57BL/6J mice (n = 8 each group) to HFD feeding with weekly CCl_4_ i.p. injections for 14 weeks and transduced with miR‐188‐5p inhibitors or NC via tail vein at different intervals (Figure S2). As shown in Figure S3A,C, the expression of miR‐188‐5p was significantly up‐regulated in the livers of mice induced with HFD + CCl_4_ but significantly reduced after the inhibition of miR‐188‐5p. Upon miR‐188‐5p inhibition in mice on HFD feeding and CCL_4_ injections, a moderate resistance to diet‐induced weight gain but moderate increase in weight was observed compared to the HFD + CCl_4_‐induced mice (Figure 4A‐C). However, there was no significant change in the visceral fat weight or ratio of visceral fat to body weight after the inhibition of miR‐188‐5p (Figure 4E,F). Moreover, there was a mild decrease in the liver to body weight ratio in the mice group administered with miR‐188‐5p inhibitors; however, it was not significant (Figure 4D,G,H).

**FIGURE 5:**
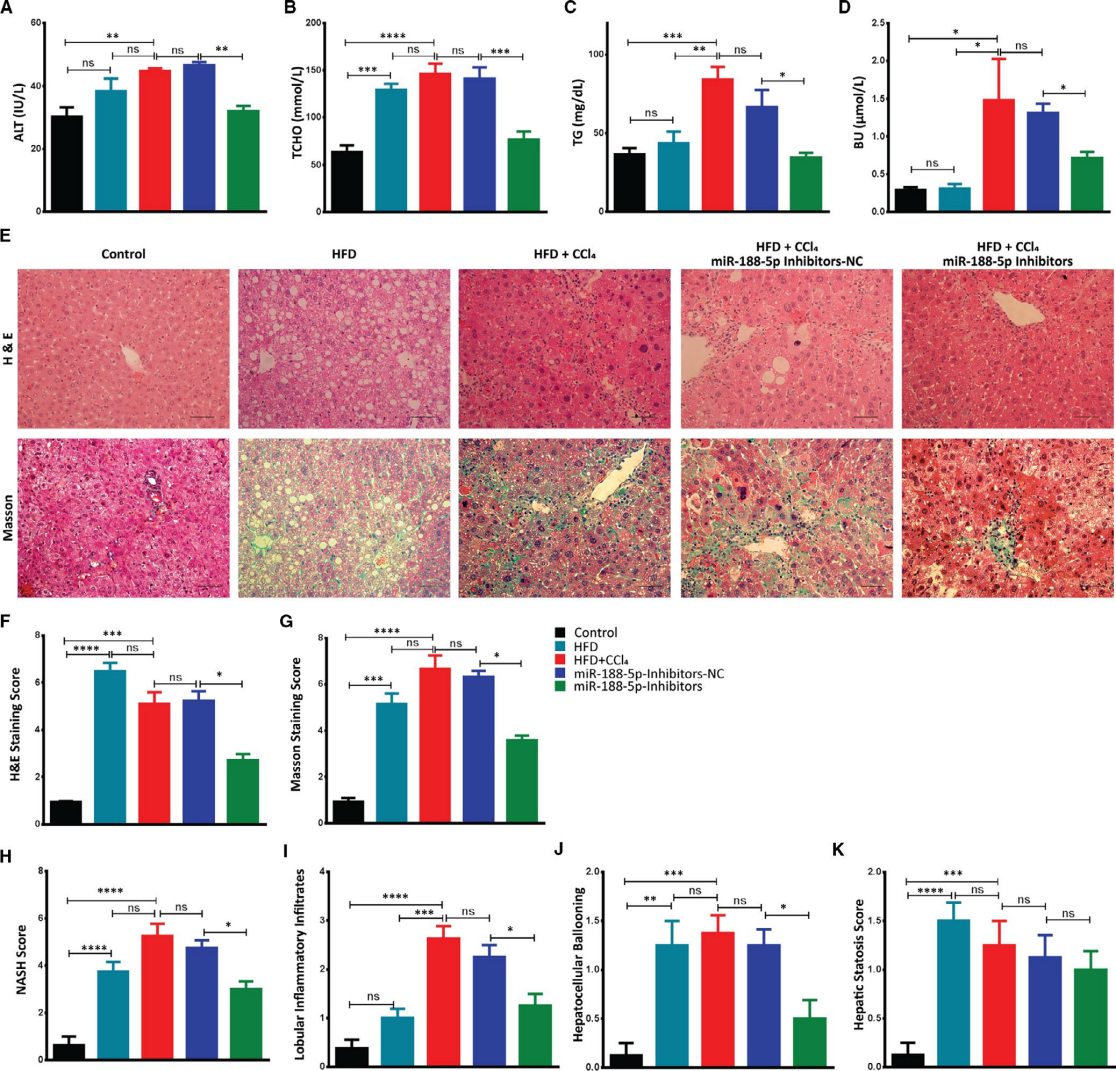
Inhibition of miR‐188‐5p rehabilitates the metabolic and histological features in HFD + CCl4‐induced in vivo hepatic fibrosis model. A‐D, Bar graph showing the serum ALT (IU/L) (A), TCHO (mmol/L) (B) TG (mg/dL) (C) and BU (μmol/L) (D) in different animal groups used for in vivo studies. E, H&E (scale bar 50 µm) and Masson staining (scale bar 50 µm) of liver specimens showing microscopic images of each animal group. F, Quantification of HE stained area showed abnormal morphological changes in the livers. G, Quantification of Masson stained area showed accumulation of collagen in the livers of different animal groups. H‐K, NASH activity score showing the level of fibrosis (H), score for liver inflammatory infiltrates (I), score for hepatocyte ballooning (J) and score for hepatic steatosis (K) in the in vivo experimental groups (n = 8 each group). Values represent as mean ± SEM. *P  < 0.05, **P  < 0.01, ***P  < 0.001 and ****P  < 0.0001

Similarly, fasting blood glucose was significantly lower in miR‐188‐5p‐inhibitor mice group (Figure 4I,K). It suggests that inhibition of miR‐188‐5p protects mice from HFD + CCl_4_‐induced insulin resistance. Accordingly, glucose tolerance test (ipGTT) and insulin tolerance test (ipITT) shown that miR‐188‐5p‐inhibitor mice showed improved glucose tolerance and boosted insulin sensitivity (Figure 4I‐L). Likewise, serum analysis showed that mice in the miR‐188‐5p inhibitors group alleviated the serum fibrosis markers, that is ALT, TCHO, TG and BU compared to miR‐NC group (Figure 5A‐D).

In addition, representative images of H&E and Masson Trichrome staining exhibited that mice in the HFD + CCl_4_ and miR‐NC groups developed severe steatosis, hepatocyte ballooning, inflammatory infiltrates and collagen deposition, and aggravated the overall NASH score (Figure 5E‐K). However, NASH score and those morphological changes were remarkably decreased in the miR‐188‐5p inhibitor group, compared with the miR‐NC groups (Figure 5E‐K). Together, these results indicated that the inhibition of miR‐188‐5p exhibited anti‐fibrotic effect by significantly improving the HFD + CCl_4_‐induced hepatic steatosis, inflammation and lesions associated with NASH.

### 3.5 Inhibition of miR‐188‐5p expression reduces the HFD + CCL_4_‐induced NASH‐associated pro‐fibrotic factors and pro‐inflammatory cytokines in liver tissue

We next followed up on elevated fibrosis markers and hepatic damage examined in the HFD + CCl_4_‐induced liver injury during serum and liver histopathology analysis, respectively, by examining the expression profile of liver inflammatory cytokines and pro‐fibrogenic genes. The present study determined that HFD + CCl_4_ enhanced the inflammatory infiltrates and degree of fibrosis in the liver. However, it was significantly decreased after the inhibition of miR‐188‐5p. Therefore, we probed into the regulatory role of miR‐188‐5p during the process of fibrogenesis by modulating pro‐inflammatory and pro‐fibrotic genes.

**FIGURE 6:**
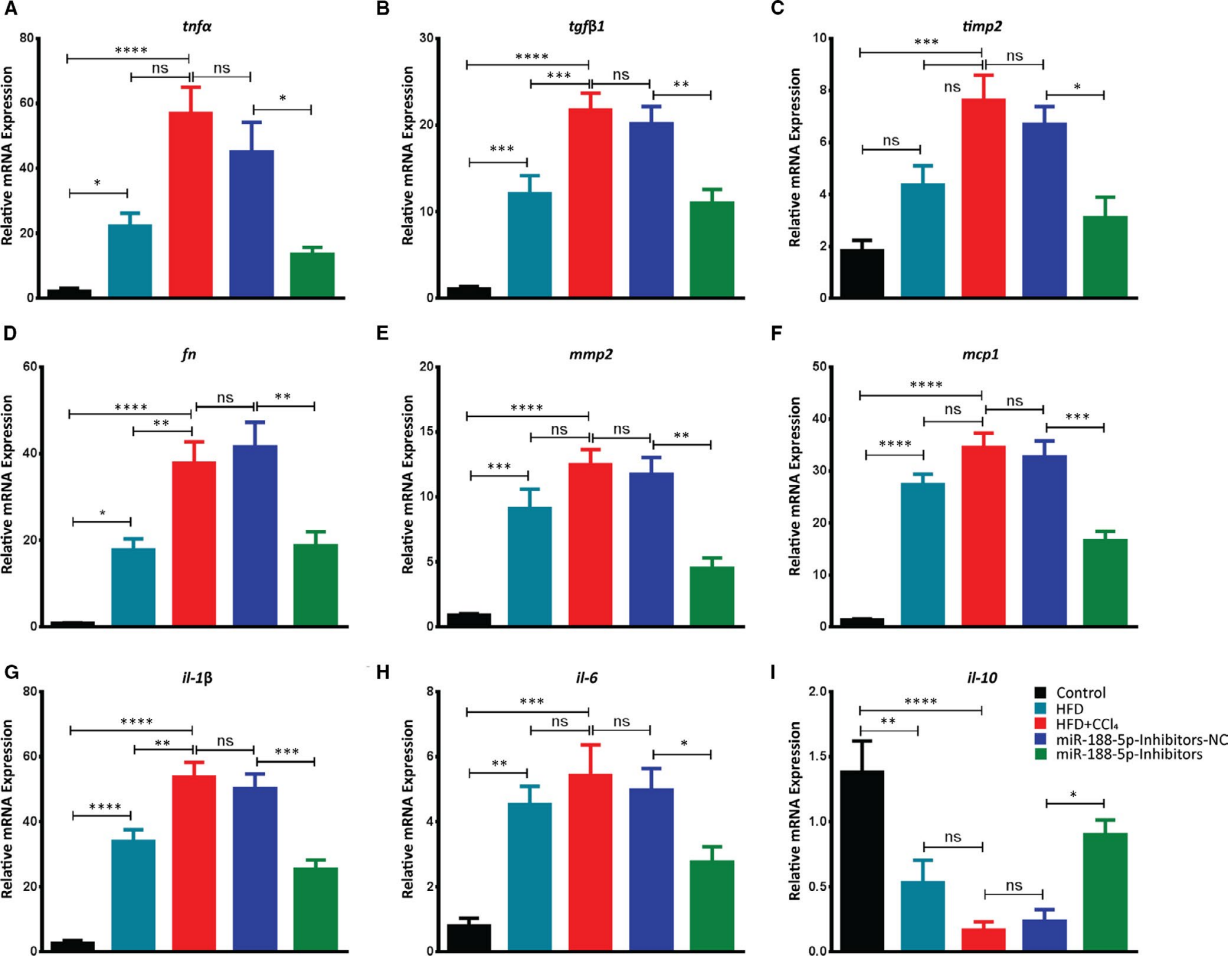
Inhibition of miR‐188‐5p down‐regulates the pro‐fibrotic and pro‐inflammatory genes in HFD + CCl_4_‐induced hepatic fibrosis. Bar graphs representing the quantitative analysis of the mRNA expression of pro‐fibrotic and pro‐inflammatory genes, *tnfα* (A), *tgfβ1*(B), *timp2* (C), *fn* (D), *mmp2* (E), *mcp1* (F), *il‐1β* (G), *il‐6* (H) and anti‐inflammatory *il‐10* (I) in the livers of in vivo mice groupsused to study the effects of miR‐188‐5p inhibition on the HFD + CCl4‐induced hepatic fibrosis (n = 8 each group). Values represent as mean  ±  SEM. *P < 0.05, **P  < 0.01, ***P < 0.001 and ****P < 0.0001

In miR‐188‐5p inhibitor group, the relative expression of pro‐fibrotic genes *tnfα*, *tgfβ*, *timp2* and *fn* significantly decreased compared to the inhibitors‐NC control group (Figure 6A‐D). Moreover, ECM remodelling gene *mmp2* which was significantly increased in HFD + CCl_4_ group was also significantly reduced after the inhibition of miR‐188‐5p (Figure 6E). Similarly, HFD + CCl_4_ significantly increased the expression level of pro‐inflammatory *mcp1, IL‐1β* and *il‐6*. However, inhibition of miR‐188‐5p significantly reduced the expression of these pro‐inflammatory genes (Figure 6F‐H). We further validated the expression level of anti‐inflammatory *il‐10* that was significantly decreased in the HFD + CCl_4_, but inhibition of miR‐188‐5p significantly increased its expression (Figure 6I). Together, these findings show that inhibition of miR‐188‐5p significantly reduces the severity of HF in HFD + CCl_4_‐induced mice showing severe NASH in term of higher pro‐fibrotic and pro‐inflammatory changes in gene expression as compared to HFD.

### 3.6 Inhibition of miR‐188‐5p expression alleviates the HF by down‐regulating the HSC activation and proliferation markers in HFD + CCl_4_‐induced liver fibrosis through PTEN/AKT pathway

**FIGURE 7:**
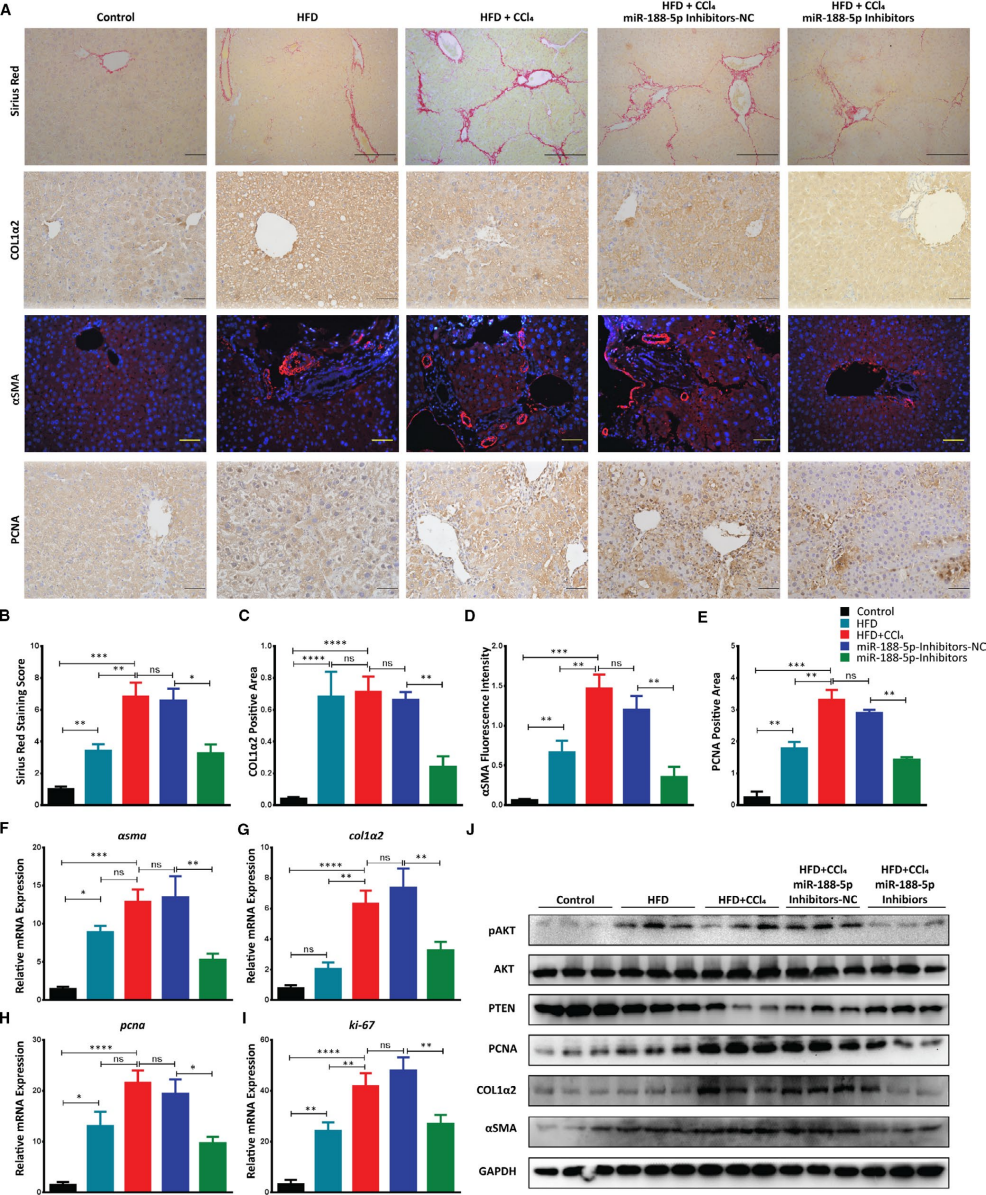
Inhibition of miR‐188‐5p reduces the severity of HF and down‐regulates the activation and proliferation of HSCs in HFD + CCl_4_‐induced HF model through the PTEN/AKT pathway. A, Sirius red staining (scale bar 100 µm), IHC staining of Col1α2 (scale bar 50 µm), IF staining of α‐SMA (scale bar 50 µm) and IHC staining of PCNA (scale bar 50 µm) in liver tissues from different mice groups used to study the effect of miR‐188‐5p inhibition on HFD + CCl_4_‐induced HF in vivo. B, Quantification of Sirius red‐stained area showed accumulation of collagen in the livers. C, Quantification of COL1α2 positive area. D, Quantification of α‐SMA fluorescence intensity. E, Quantification of PCNA positive area in the livers of different animal groups. F, Relative mRNA expression of *αsma*. G, Relative mRNA expression of *col1α2*. H, Relative mRNA expression of *pcna*. I, Relative mRNA expression of *ki‐67* in liver tissues from different mice groups used to study the effect of miR‐188‐5p inhibition on HFD + CCl_4_ induced HF in vivo. J, Western blot analysis showing the inhibitory effects of miR‐188‐5p‐inhibitors on HSC activation marker α‐SMA and COL1α2, proliferation marker PCNA and PTEN/AKT pathway in whole liver lysate from different mice groups used to study the effect of miR‐188‐5p inhibition on HFD + CCl_4_‐induced HF in vivo (n = 8 each group). Values represent as mean  ±  SEM. *P  < 0.05, **P  < 0.01, ***P  < 0.001 and ****P  < 0.0001. Abbreviation: HF, hepatic fibrosis

Activation and proliferation of HSCs are correlated with the progression and severity of HF. Above findings bought us the regulatory role of miR‐188‐5p in the activation and proliferation of HSCs. Therefore, we further examined the inhibitory role of miR‐188‐5p on HSC activation and proliferation in HFD + CCl_4_‐induced HF model. Sirius red staining showed a significant increase of the collagen accumulation in HFD, HFD + CCl4 and miR‐188‐5p NC group (Figure 7A,B). In order to examine the expression of HSC activation markers Col1α2 and α‐SMA, we performed IHC and IF, respectively. Image J analysis of stained slides showed the significant decrease in the expression of HSC activation markers after the inhibition of miR‐188‐5p (Figure 7A,C,D). Similarly, IHC analysis of HSC proliferation marker PCNA showed significant decrease in the in vivo miR‐188‐5p‐inhibitors group (Figure 7A,E). Interestingly, Sirius red staining showed a significant decrease in the accumulation of collagen after the inhibition of miR‐188‐5p (Figure 7A,B), thus representing inhibition of miR‐188‐5p can significantly decrease the severity of fibrosis in vivo. To further validate the role of miR‐188‐5p inhibition in vivo, we performed RT‐qPCR to examine the expression of HSC activation markers (*αsma* and *col1α2*) and HSC proliferation markers (*pcna* and *ki‐67*). As expected, the expression of HSC activation and proliferation markers, which was significantly increased in HFD + CCl4 group, was significantly down‐regulated after the inhibition of miR‐188‐5p (Figure 7F‐I).

Furthermore, immunoblotting analysis showed that HSC activation markers α‐SMA and COL1A2 were significantly down‐regulated in liver samples after the inhibition of miR‐188‐5p (Figure 7J). A similar trend was observed for the HSC proliferation marker PCNA that was also significantly decreased after the inhibition of miR‐188‐5p. Remarkably, inhibition of miR‐188‐5p led to a significant increase in expression of its target gene PTEN at both transcriptional and translational level (Figure 7J, Figure S3B,C). This increase in the PTEN expression after the inhibition of miR‐188‐5p led to the down‐regulation of AKT pathway (Figure 7J). Thus, we observed a significant decrease in the activation and proliferation markers after the inhibition of miR‐188‐5p. Collectively, these results revealed that inhibition of miR‐188‐5p led to the down‐regulation of HSC activation and proliferation markers and severity of HF through PTEN/AKT pathway.

## 4 DISCUSSION

HF characterizes a major health problem worldwide. It is considered as a scarring process that is associated with excessive deposition of ECM in liver. HSCs are the primary effector cells for the deposition of ECM in fibrotic liver.^33^ This progressive fibrotic response is characterized by activation and proliferation of HSCs, and aberrant expression of cellular transcriptional factors.^34^ Ample evidence has revealed that ncRNAs, especially numerous miRNAs, function as critical regulators in activation and proliferation of HSCs, and regulate liver fibrosis.^9^, ^35^, ^36^, ^37^ However, the function and underlying mechanism of miR‐188‐5p in HSC activation, proliferation and liver fibrosis remain unknown. In the current study, we investigated the potential role of miR‐188‐5p in activation and proliferation of HSC and NASH‐associated liver fibrosis.

Our results demonstrated that miR‐188‐5p is highly expressed in HFD‐induced murine models of NASH and TGF‐β1‐induced human LX‐2 cells compared to normal liver tissues and LX‐2 cell line, respectively. However, it has not been studied how TGF‐β1 induces the maturation and the expression of miR‐188‐5p and several other dysregulated miRNAs. Some studies have probed that TGFβ signalling links with Drosha/DGCR8 complex in SMAD‐dependent manner and modulates the maturation of several miRNAs.^38^, ^39^, ^40^ Therefore, we believe it needs further studies to determine which TGF‐β1 downstream pathways are involved in the biogenesis and maturation of miRNAs, including miR‐188‐5p. Moreover, the human NAFLD liver specimens showed a similar trend. However, the study is limited to fewer human samples and use of CHB subjects as NAFLD control. Despite this, our results are in accordance with previous finding suggesting that expression of miR‐188‐5p is decreased in tumour tissues of HCC patients, while no difference was observed among samples from Hepatitis B&C virus positive patients.^41^ Therefore, our finding reached an agreement with previous findings that miR‐188‐5p is up‐regulated in CCl_4_‐induced HF,^15^ tensile‐strained HSCs,^30^ and liquid biopsies from alcohol‐induced liver cirrhosis and toxic doses of acetaminophen (APAP) liver injury patients. Therefore, inhibition of miR‐188‐5p can play a regulatory role in HF. Several other reports also demonstrated that miR‐188‐5p participated in cellular processes of several diseases, including gastric cancer,^22^ retinoblastoma,^26^ non‐small‐cell lung cancer,^25^ prostate cancer,^21^ glioma,^27^ hepatocellular carcinoma,^24^ Alzheimer's Disease,^28^ rheumatoid arthritis^23^ and diabetic kidney disease.^42^

Furthermore, we found that up‐regulation of miR‐188‐5p resulted in the up‐regulation of HSC activation markers α‐SMA and type I collagen. Whereas, inhibition of miR‐188‐5p lead to the down‐regulation of these HSC activation markers. Notably, bioinformatics tools suggest that there is no direct binding between miR‐188‐5p and these markers. However, there is an indirect mechanism involved in the regulation of HSC activation markers. Similar to our results, a study by Ruedel et al^23^ also demonstrated that miR‐188‐5p regulates the expression of type I collagen in an indirect mechanism.

It has been well documented that PTEN negatively regulates the process of liver fibrosis.^43^ Several miRNAs have been reported to directly bind with PTEN to regulate its expression and play critical roles in the activation and proliferation of HSCs.^44^, ^45^ We observed that PTEN expression was negatively regulated by miR‐188‐5p at the post‐transcriptional level, via a specific 3′ UTR target site, which supported that miR‐188‐5p expression increased during liver fibrosis, and its inhibition could play a regulatory role in the activation and proliferation of HSCs. Notably, inhibition of miR‐188‐5p significantly led to the suppression of HSC activation and proliferation in vitro and in vivo via up‐regulation of PTEN expression through PI3K/AKT pathway. Owing to the restoration of PTEN, in vivo suppression of miR‐188‐5p contributed to the suppression of pro‐fibrotic and pro‐inflammatory genes in HFD + CCl_4_‐induced mouse model. Whereas, our in vitro results suggested there was no significant change in the expression of miR‐188‐5p after the overexpression of PTEN in LX‐2 cells (data not shown) indicating that another upstream pathway is involved in the regulation of miR‐188‐5p expression which needs further investigation. However, we also observed the suppression of miR‐188‐5p expression after the transfection of miR‐188‐5p inhibitors, which might result from miRNA sequestering and degradation.^46^, ^47^, ^48^ Overall, our study identified miR‐188‐5p as an important regulator of HSC cell activation and proliferation, and emphasized a critical role of this miRNA in mediating HF, at least in part, via PTEN/PI3K/AKT.

Previous studies have demonstrated that altered miRNA expression is closely associated with liver fibrosis.^12^ However, the functions of miRNA may vary. Studies have shown that several miRNAs can function as pro‐fibrotic, while other function as anti‐fibrotic.^31^, ^49^, ^50^, ^51^ In the current study, miR‐188‐5p was identified to up‐regulate in HF. Furthermore, its up‐regulation is associated with high expression of pro‐fibrotic and pro‐inflammatory genes, which strongly suggests a potential role of miR‐188‐5p in the progression of liver fibrosis. Hyun et al^15^, ^16^ took advantage of microarray technology to identify that miR‐188‐5p was up‐regulated in CCl4‐induced mice model. Similarly, the miR‐188‐5p expression was significantly up‐regulated in HSCs in response to tensile strain representing the mechanical response of HSCs to portal hypertension.^30^ In line with this, our results indicated that the up‐regulation of miR‐188‐5p in HF may facilitate the activation and proliferation of HSC. However, its biological role in HF remains poorly understood. In this report, we tried to figure out the potential role of miR‐188‐5p in HSCs activation and proliferation, both in vitro and in vivo.

Activation of HSCs is a tightly orchestrated process that includes several functional changes, such as increased proliferation and synthesis of ECM components.^52^ To date, numerous miRNAs have been shown to regulate proliferation of HSCs, such as miR‐150,^53^ miR‐122,^54^ miR‐15/16^55^ and miR‐335^56^ while other miRNAs, such as miR‐133 and miR‐29, are implicated in HF by regulating production of ECM components.^57^ As discussed earlier, miR‐188‐5p regulated HSC activation and proliferation by regulating PTEN expression. The role of PTEN in liver fibrosis has been well documented.^32^, ^58^, ^59^ Reduced PTEN expression in liver fibrosis leads to PIP3 accumulation resulting in activation of PI3kinase followed by phosphorylation of AKT. AKT is a pro‐cell survival protein that is well documented for its role in HSC proliferation and hepatic injury.^32^, ^60^ Several miRNAs play a regulatory role in PI3K/AKT pathway by targeting the PTEN. For example, miR‐140‐3p mediates TGFβ‐1‐induced HSC‐T6 cell activation and the inhibition of miR‐140‐3p down‐regulated cell proliferation and fibrogenesis in TGF‐β1‐induced HSC‐T6 cells.^58^ Here, we showed that miR‐188‐5p directly suppressed PTEN expression and consequently increased the level of α‐SMA, Col1α2, and PCNA in HSCs through PI3K/AKT pathway. Therefore, our findings suggest that miR‐188‐5p promotes α‐SMA expression and cell proliferation by targeting the PTEN/PI3K/AKT signalling pathway. As shown, inhibition of miR‐188‐5p reduced the level of α‐SMA, Col1α2 and PCNA in TGF‐β1‐induced LX‐2 cells.

Many researchers have focused on the development of a definite model to study NASH. Recently, combination of diet (HFD) and chemical (CCl_4_)‐induced NASH model has gained popularity because of its ability to develop fatty liver disease rapid.^19^, ^20^ Consistent with these studies, our HFD + CCl_4_ animal model also showed high degree of fibrosis compared to the NASH model induced with HFD only. Inhibition of miR‐188‐5p in HFD + CCl_4_‐induced model revealed a significant decrease in pro‐fibrotic and pro‐inflammatory genes in HFD + CCl4‐induced liver fibrosis. Therefore, we focused on exploring whether miR‐188‐5p promoted fibrosis by regulating the activation and proliferation of HSCs.

**FIGURE 8:**
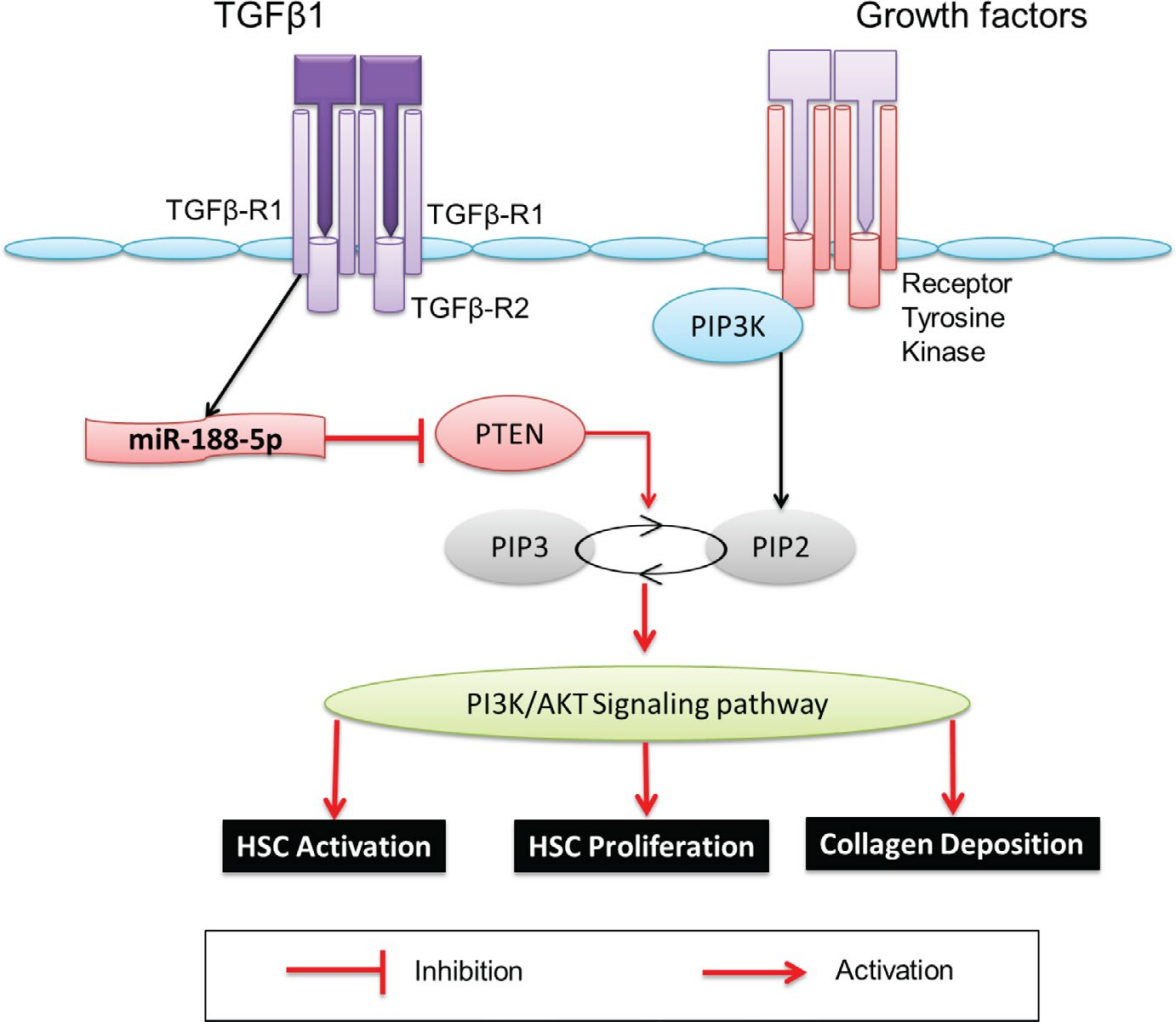
Schematic representation of miR‐188‐5p‐dependent regulation of the PTEN/AKT pathway involved in the progression of HF. This diagram shows that during the process of HF, miR‐188‐5p is up‐regulated and it inhibits the expression PTEN that leads to the activation of the PI3K/AKT pathway. This activation of the PI3K/AKT pathway leads to the activation and proliferation of HSC, collagen deposition, and progression of HF. Abbreviation: HF, hepatic fibrosis

In summary, we found that miR‐188‐5p expression is significantly up‐regulated during HSC activation and HF. Knockdown of miR‐188‐5p inhibits the expression of pro‐fibrotic and pro‐inflammatory markers and cell proliferation in HSCs by targeting PTEN through inhibiting the PTEN/PI3K/AKT pathway (Figure 8). Moreover, miR‐188‐5p inhibition prevented HF in mice. Therefore, our findings provide new insights into the cellular mechanisms by urging that miR‐188‐5p alleviates liver fibrosis by regulating the PTEN/PI3K/AKT signalling pathway.

## CONFLICT OF INTERESTS

The authors declare that there is no conflict of interests.

## AUTHOR CONTRIBUTIONS

Farooq Riaz: Conceptualization (lead); Writing‐original draft (lead); Writing‐review & editing (lead). Qian Chen: Methodology (supporting); Resources (supporting). Kaikai Lu: Methodology (supporting). Ezra Kombo Osoro: Methodology (supporting). Litao Wu: Resources (supporting). Lina Feng: Methodology (supporting). Rong Zhao: Methodology (supporting). Luyun Yang: Methodology (supporting). Yimeng Zhou: Methodology (supporting). YingLi He: Methodology (supporting); Resources (supporting). Li Zhu: Methodology (supporting); Resources (supporting). Xiaojuan Du: Methodology (supporting). Muhammad Sadiq: Methodology (supporting). Xudong Yang: Methodology (supporting). Dongmin Li: Funding acquisition (lead); Supervision (lead); Writing‐review & editing (supporting).

## Supporting information

Supplementary Material
