# Supplementary material for: Inhibition of miR‐188‐5p alleviates hepatic fibrosis by significantly reducing the activation and proliferation of HSCs through PTEN/PI3K/AKT pathway
Source: J Cell Mol Med. 2021 Mar 10;25(8):4073–87. doi: 10.1111/jcmm.16376 (PMC8051718; doi:10.1111/jcmm.16376)
Supplement: Supplementary file 1 — Supplementary Material [file JCMM-25-4073-s001.docx]

**Inhibition of miR-188-5p** **alleviates Hepatic Fibrosis by significantly reducing the activation and proliferation of HSCs through PTEN/PI3K/AKT pathway**

Farooq Riaz^1,2^, Qian Chen^1,2^, Kaikai Lu^1,2^, Ezra Kombo Osoro^1,2^, Litao Wu^1,2^, Lina Feng^1,2^, Rong Zhao^1,2^, Luyun Yang^1,2^, Yimeng Zhou^1,2^, Yingli He^3^, Li Zhu^3^, [Xiaojuan Du](https://www.researchgate.net/scientific-contributions/2141816801_Xiaojuan_Du)^1,2^, Muhammad Sadiq^1,2^, Xudong Yang^1,2^ and Dongmin Li^1,2*^

^1^Department of Biochemistry and Molecular Biology, School of Basic Medical Sciences, Xi’an Jiaotong University Health Science Center, Xi’an, Shaanxi 710061, P.R. China

^2^Key Laboratory of Environment and Genes Related to Diseases, Ministry of Education, Xi’an, Shaanxi 710061, P.R. China

^3^Department of Infectious Diseases, First Affiliated Hospital of Xi’an Jiaotong University, Xi’an 710061, China.

^*^Address correspondence to this author at the Department of Biochemistry and Molecular Biology, School of Basic Medical Sciences, Xi’an Jiaotong University Health Science Center, Xi’an, Shaanxi 710061, P.R. China

Tel: +86-029-82657013

E-mail: lidongm@mail.xjtu.edu.cn

**Supporting Material**

**Materials and Methods:**

**Cell culture**

Immortalized human HSC cell line (LX-2) was maintained in Dulbecco’s modified Eagle’s medium (DMEM) high glucose (HyClone, Cat# SH30022) complemented with 10% fetal bovine serum (FBS, Gibco, Cat# 10270) and 100 IU/mL penicillin-streptomycin. Cells were maintained in humid incubator containing 5% CO_2_ for 48h at 37°C.

**TGF-β1 activation of LX-2 cells**

LX-2 cells were seeded in 6-well plates at a density of 4×10^5^ cells/well. Cells were starved for 6h prior to treatments in serum-free DMEM media, following which cells were activated with different doses (2.5, 5 and 10 ng/ml) of human TGF-β (Peprotech, Cat# 100-21) for activation of HSCs. After 48h of TGF-β treatment, the cells were collected for subsequent experiments.

**Cell transfection**

Following the 24h incubation of LX-2 cells cultured in 6-well plates, cells were starved in serum-free DMEM for 6h and then transfected with miR-188-5p inhibitors, miR-188-5p mimics, siPTEN or their respectively negative controls (NC) at a final concentration of 80 nM/L. All the miRNAs and siRNAs were synthesized by Shanghai GenePharma Co., Ltd., (China). All the oligonucleotides used in this study are listed in (Supplementary Table S1). Over-expressing human PTEN (NM_000314) or control pcDNA3.1 plasmid (purchased from FENGHUISHENGWU, China) were transfected into LX-2 cells at the concentration of 4 μg/ml. All these oligonucleotides and plasmids were diluted in Opti-MEM (Invitrogen) and transfection was performed with Lipofectamine® 2000 (Invitrogen, Cat# 11668019) according to the manufacturer’s instruction. After 6h post-transfection, culture media was replaced with fresh DMEM/high glucose culture media containing 2% FBS for an additional 48h and then harvested for subsequent experiments.

**RT-qPCR**

Total RNA was extracted from LX-2 cells or animal tissues using Trizol^®^ reagent (Invitrogen). Quantity and integrity of total RNA was determined by NanoDrop-2000 (Thermo Fisher Scientific). Template cDNA synthesis was performed using miScript-II RT Kit (Qiagen, Cat# 218161) or the Revert-Aid First-Strand cDNA synthesis kit (Thermo Fischer Scientific, Cat# K1622) following the manufacturer’s instructions. To quantify miR-188-5p and mRNA expression levels, quantitative real-time PCR (RT-qPCR) was performed using the miScript SYBR Green PCR Kit (Qiagen, Cat# 218073) or FastStart Universal SYBR Green Master (ROX) Kit (Roche, Cat# 13396700) on the Stratagene Mx3005P real-time thermal cycler (Agilent Technologies, Santa Clara, CA). Primers used in this study are listed in Supplementary Table S1. All the RT-qPCR experiments were performed in triplicate and the relative change in expression level analyzed using the ∆∆C_t_ method. Results were normalized by the U6 or GAPDH mRNA as endogenous control.

**Western blotting**

Total protein from liver tissues or LX-2 cells was extracted by homogenizing tissue or cells in RIPA buffer (Beyotime, Cat# P0013). Protein concentration was determined by using BCA Protein Assay Kit (Tiangen, Cat# PA115). Then, 20 μg of total protein per sample was separated by 10% sodium dodecyl sulfate–polyacrylamide (SDS-PAGE) gel electrophoresis, followed by transferring to a polyvineyledene fluoride membrance (PVDF) membrane (Millipore). After blocking with 5% skim-milk, the membranes were incubated overnight with the appropriate primary antibodies at 4°C. After washing with TBST, blots were incubated with corresponding HRP-conjugated secondary antibodies to bind with primary antibodies at RT for 2h. The protein bands were visualized with enhanced chemiluminescence (ECL) (Millipore, WBKLS0100). GAPDH was used as the internal control to normalize the results. All the antibodies used in this study are listed in Supplementary Table S2.

**Bioinformatics**

The putative miR-188-5p target genes were selected from the predictive outcomes by three widely used algorithms TargetScan 7.2 (http://www.targetscan.org), MIRDB (http://mirdb.org/) and PicTar (http://pictar.mdc-berlin.de/).

**Dual luciferase assay**

Based on the bioinformatics analysis for miR-188-5p, the oligonucleotides for the predicted binding site region (3’UTR) targeting wild type (WT) hsa-PTEN mRNA or the corresponding mutated region (Mut) (Figure 3A) were synthesized, and annealed using annealing buffer (Beyotime, D0251) in a thermal cycler according to the manufacturer’s instruction. WT and Mut oligonucleotides sequences used in this study are mentioned in Supplementary Table S1. Later, the annealed product for WT or Mut PTEN was cloned into pmirGLO Dual-Luciferase Vector (Promega, Given by associate professor Congshan Jiang). An empty pmirGLO vector was used as negative control. HEK293T cultured in DMEM/high glucose and 10% FBS were seeded into 96-well plates at a density of 2 × 10^3^ cells/well, and incubated at 37°C for 24h in an incubator with 5% CO_2_ humidified atmosphere. Then, the cells were co-transfected with pmir-PTEN (WT or mutant) and miR-188-5p mimics using Lipofectamine^®^ 2000. After 48h post-transfection, cells were harvested and tested for luciferase activities by a dual luciferase reporter assay system (Promega, Cat# E1910) with the Infinite M1000, TECAN microplate reader.

**Cell proliferation**

The cell proliferation activity of HSCs was determined in human LX-2 cells using Cell Counting Kit-8 (CCK-8) (Beyotime, China). Briefly, the LX-2 cells were plated in a 96 well plate at a density of 2 × 10^3^ cells/well for 24h. After starvation for 4h, cells were transfected with miR-188-5p mimics, miR-188-5p inhibitors or respective miR-NC for 6h. Later, the culture medium was replaced with the fresh medium and the cells were cultivated at different time intervals to measure the proliferation activity according to the manufacturer’s protocol.

**Glucose and insulin tolerance tests**

Animals were fasted for overnight before performing the intra-peritoneal insulin tolerance test (ipITT) and intra-peritoneal glucose tolerance test (ipGTT) at 12^th^ and 13^th^ week, respectively. Briefly, 0.75 U/Kg of insulin or 150 μl of 20 % glucose was administered in mice after measuring the baseline glucose level. After administering the insulin or glucose, blood glucose was measured at 15, 30, 60 and 90 min.

**Serum analysis**

Blood samples were collected through cardiac puncture. Serum was collected from blood samples by centrifuging collected blood samples at 3000 rpm for 10 min at 4°C. Subsequently, supernatant was centrifuged at 16,000 rpm for 10 min at 4°C to remove any cell debris. The resulting serum was then stored at −80°C for further analysis. Serum biochemistry analysis was analyzed using an automated bio-analyzer (Sebia, France) from the Second Affiliated Hospital of Xi’an Jiaotong University.

**Histopathological analysis:**

Formalin-fixed paraffin embedded (FFPE) liver specimens were processed for histopathological analysis using the standard protocols. Specimens were sectioned at 5 μm intervals and stained with Haematoxylin and Eosin (H&E) and NAFLD Activity Score (NAS) which includes hepatic steatosis, inflammatory infiltrates and hepatocellular ballooning. Furthermore, Masson’s Trichome or Sirius Red stainings were performed to assess liver fibrosis by collagen deposition. All slides were visualized under inverted microscope (Olympus) at low (100 X), medium (200 X) and high (400 X) magnification. ImageJ was used for histopathological data analysis.

**Immunohistochemistry (IHC)**

For immunohistochemistry (IHC), FFPE sections were hydrated and heated in 10 mM sodium citrate buffer (pH 6.0) for antigen retrieval at 95°C until temperature reached 65°C followed by the use of anti-rabbit IHC kit (CW Bio, Cat# CW20355) according to the manufacturer protocol. Sections were incubated with respective primary antibodies for overnight at 4°C in a wet box. Subsequently, slides were incubated with anti-rabbit HRP conjugated Cy-3 or HRP-polymer-conjugated anti-rabbit secondary antibody for 1h and 30 minutes respectively. 3,3′-diaminobenzidine tetrachloride (DAB) was used for signal development. Nuclear counterstaining was achieved by using Haematoxylin, followed by dehydration. All the specimens were mounted using a mounting medium. Slides were viewed with microscope (Olympus). The staining intensity of each section was analyzed by ImageJ.

**Immunofluorescence (IF) studies**

LX-2 cells, seeded and treated in 24-well plates on coverslips, were fixed in 4% paraformaldehyde for 15 min at RT, permeabilized with 0.2 % TritonX-100 at RT for 20 min, and blocked with PBS containing 5 % bovine serum albumin for 1h at RT. Afterward, cells were incubated with primary antibodies overnight at 4°C. After washing with PBS for three times, cells were incubated with secondary antibody. Then cells were washed and nuclei were counterstained with DAPI for 3 min and mounted on glass slide to observe under microscope.

**BrdU incorporation assay**

To determine the HSC proliferation activity, LX-2 cells were transfected with miR-188-5p inhibitors and the NC on coverslips in 24 well plates for 48h. Four hours before the collection of cells, 10 µg/mL bromodeoxyuridine (BrdU) (MedChemExpress, CS-3028) was added to each well to incorporate into *de novo*-synthesized DNA. Cells were fixed and permeabilized as described earlier. Later, the BrdU positive cells were incubated with 1.5 M HCL for 30 min to denature genomic DNA and washed with PBS. Cells were then incubated with primary antibody, cocktail of anti-mouse BrdU and anti-rabbit PCNA at 4°C overnight. After washing with PBST, cells were incubated with respective secondary antibodies, cocktail of anti-mouse and anti-rabbit antibodies for 1h at RT. Nuclei were counterstained with DAPI and coverslips were mounted on glass slides. The fluorescence results were observed and photographed using fluorescence microscope.

**Fluorescence *in-situ* hybridization**

To detect the miR-188-5p through fluorescence *in-situ* hybridization (FISH), FISH kit (GenePharma, Shanghai, China) was used according to the manufacturer’s protocol. Briefly, FFPE specimens were incubated with Cy-3 labeled miR-188-5p probes at 37°C for overnight. Then the nuclei were counterstained with DAPI and mounted with glass coverslip to observe under microscope.

**Supplementary Figures**

**Supplementary Figure 1: TGFβ-1 activated the HSCs and induced the expression of pro-fibrotic genes *in-vitro*.**

A-C. The mRNA expression of HSC activation marker and pro-fibrotic gene *αsma* (A), *col1α2* (B) and *col3α1* (C), following HSC activation at different doses of TGFβ1 in LX-2 cells. D. Western blotting analysis showed the protein expression level of HSC activation marker and pro-fibrotic gene αSMA and COL1A2 at different doses of TGFβ1 in LX-2 cells. E-F. Quantification of protein expression of αSMA (E) and COL1A2 (F) at different doses of TGFβ1 in LX-2 cells. Values represent as mean ± SEM, **P* < 0.05, ***P* < 0.01, ****P* < 0.001 and *****P* < 0.0001.

**Supplementary Figure2: Schematic representation of the *in-vivo* experimental model.**

40 male C57BL/6J mice were randomized into five groups (n=8 mice in each group): Control group, mice were fed on normal diet; HFD group, mice were fed on HFD along with weekly 200 μl intraperitoneal (i.p.) injection of corn oil for 14 weeks; HFD+CCl_4_ group, mice were fed with HFD along with weekly i.p. administration of CCL_4_ diluted in corn oil (CCl_4_ at dose rate of 0.25 μl (0.40 μg)/g of body weight diluted in 200 μl of corn oil) for 14 weeks; HFD+CCl_4_-miR-NC group; and HFD+CCl_4_-miR-188-5p-inhibitor group. MiR-188 inhibitors or miR-NC were injected in respective groups of mice through tail vein injection at the mid of 5^th^week, start of 6^th^ and 7^th^ week. GTT and ITT were performed at 12^th^ and 13^th^ week, respectively. All mice were sacrificed at 14^th^ week.

**Supplementary Figure 3: *In-vivo* inhibition of miR-188-5p significantly inhibited the expression of miR-188-5p and up-regulated its target gene PTEN.**

A. Relative miRNA expression of miR-188-5p. B. Relative mRNA expression of pten. C. FISH images (scale bar 50 µm) representing the expression of miR-188-5p and IHC staining of PTEN (scale bar 50 µm) in the liver tissues from different mice groups used to study the effect of miR-188-5p inhibition on HFD+CCl4 induced HF *in-vivo*. n=8 each group. Values represent as mean ± SEM *P < 0.05, **P < 0.01, ***P < 0.001 and ****P < 0.0001.

**Supplementary Figure 1**


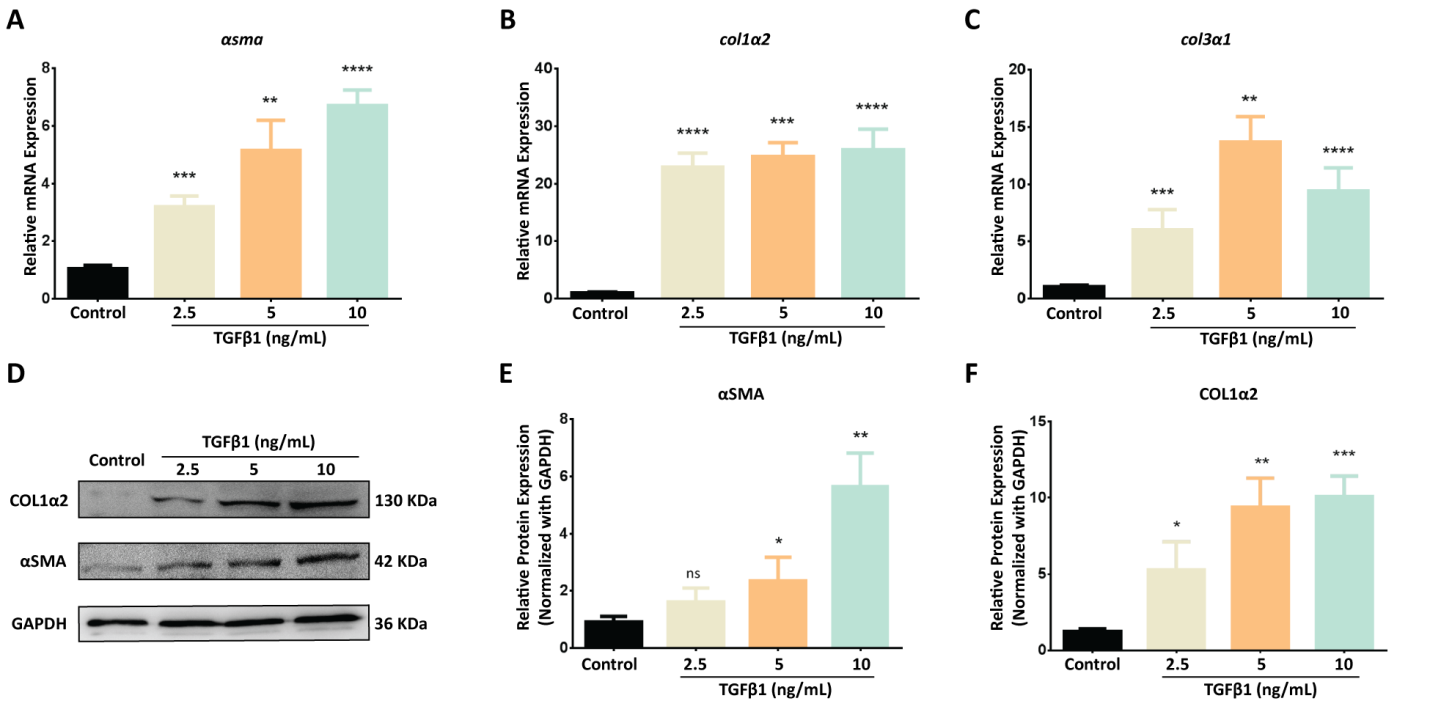
**Supplementary Figure 2**


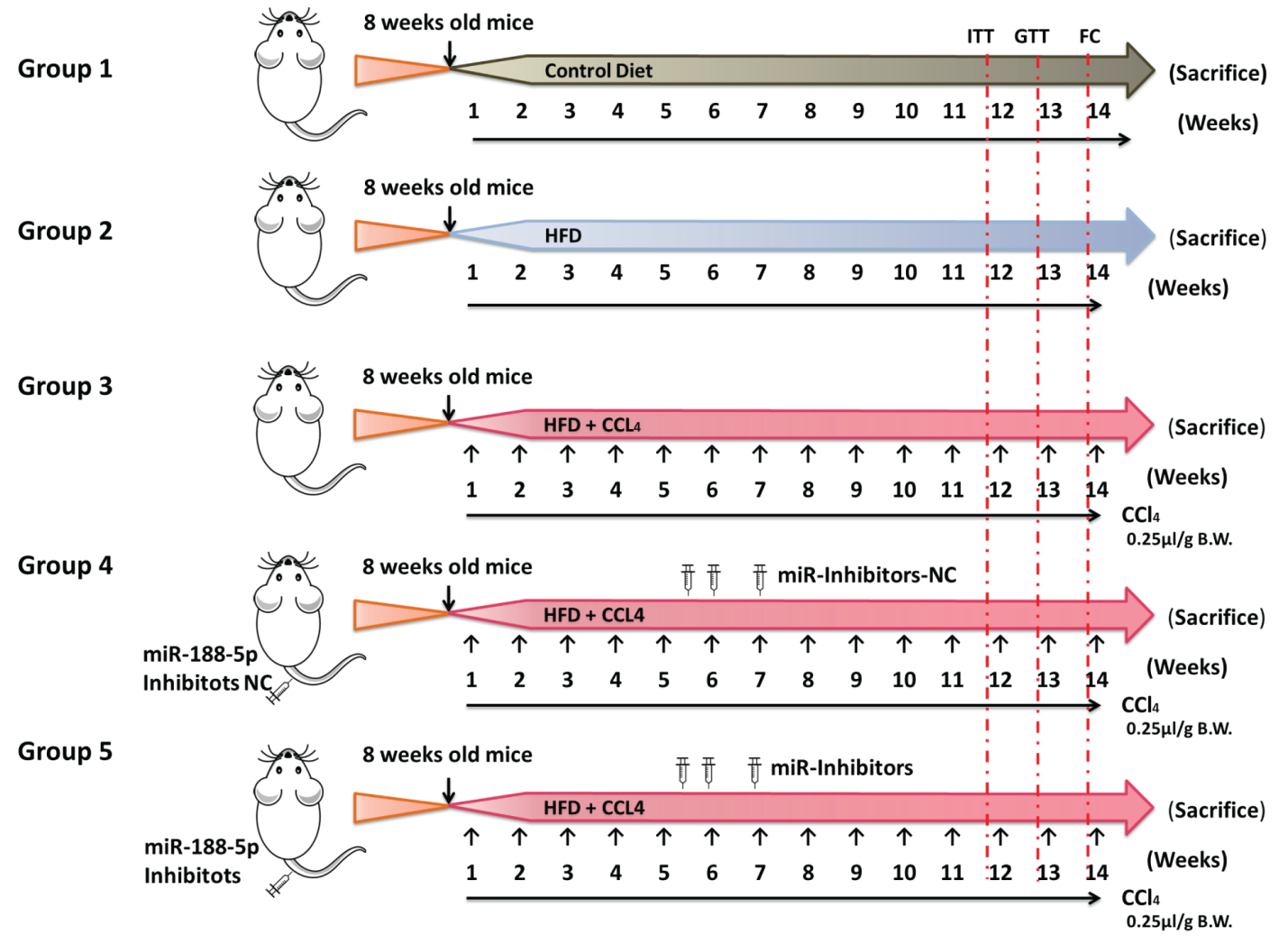


**Supplementary Figure 3**
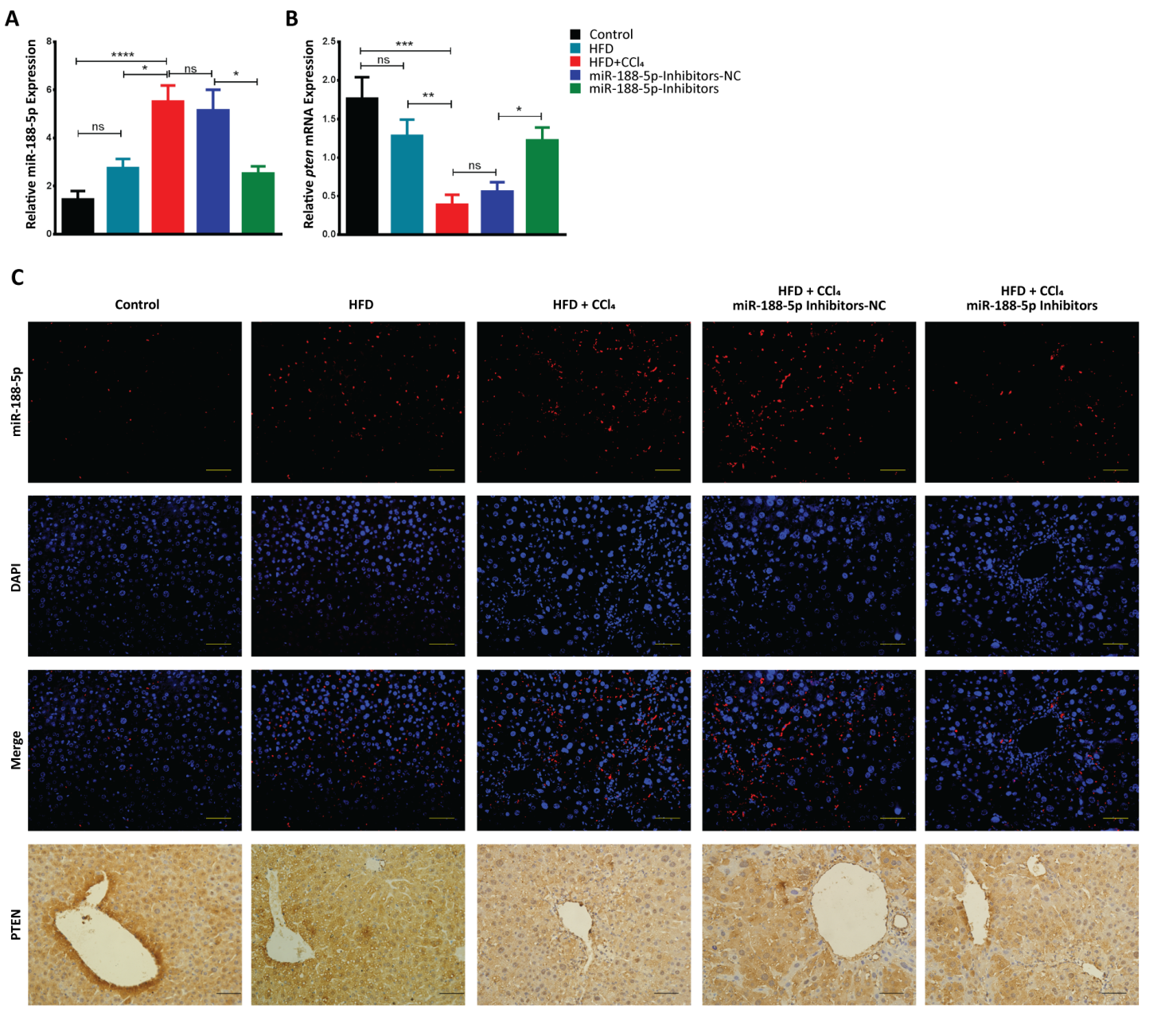


**Supplementary Table1:** List of Oligonucleotides used in this study

| **Primers** | | |
| --- | --- | --- |
| **Gene name** | **Forward Primer** | **Reverse Primer** |
| hsa-αsma | GAAGAGCATCCCACCCTGC | TAGCCACATACATGGCTGGG |
| hsa-col1α2 | TGTGGATACGCGGACTTTG | TCCATCATACTGAGCAGCAA |
| hsa-col3α1 | ACTTCTCGCTCTGCTTCATC | TTAGGAGGGCGAGTAGGAGCA |
| hsa-pten | AGACCATAACCCACCACAGC | TTTAAAAATTTGCCCCGATG |
| hsa-gapdh | GCTCTCTGCTCCTCCTGTTC | CCATGTAGTTGAGGTCAATG |
| mmu-gapdh | AGGTCGGTGTGAACGGATTTG | GGGGTCGTTGATGGCAACA |
| mmu-pten | GATGCTAGCATGACAGCCATC | GCAAGCTTTCAGACTTTTGTA |
| mmu-αsma | CCAACCGGGAGAAAATGA | CCAGACGCATGATGGCAT |
| mmu-col1α2 | TGGTCCTGATGGCAAAAC | TCCATCTTTGCCAGCAGGA |
| mmu-fn | GTCTCCTGGGAGAGGAGCA | CTGATCAGCATGGACCACT |
| mmu-tgfb | AACTATTGCTTCAGCTCC | CAGAAGTTGGCATGGTAGC |
| mmu-pcna | TACCGCTGCGACCGCAAC | ATAGTCTGAAACTTTCTC |
| mmu-ki-67 | CAACTTTGGTGATTCCATT | ATTAGGAGGCAAGTTTTCA |
| mmu-mmp2 | CCCTTCACTTTCCTGGGCAA | AAGTTCTTGGTGTAGGTGTA |
| mmu-il-10 | GGCGCTGTCATCGATTTCT | TTTGACATCTTCATCAACT |
| mmu-il-6 | CAATCTTAATAAGGTTTCC | TTGAGACTCATGGGAAAATC |
| mmu-timp2 | TGAACCACAGGTACCAGA | TCTTGATGCAGGCGAAGAA |
| mmu-tnfa | ATCGGTCCCCAAAGGGAT | TGTCTTTGAGATCCATGC |
| mmu-mcp1 | GATCCCAATGAGTAGGCT | GATGCATTAGCTTCAGAT |
| mmu-il-1 | CAGGCAGTATCACTCATT | GGAGCCTGTAGTGCAGTT |
| miR188-5p | Qiagen (Cat# MS00001757) |  |
| snRNA RNU6B | Qiagen (Cat# MS00033740) |  |
| **Oligonucleotides for transfection** | | |
| **Target** | **Sense** | **Antisense** |
| miR-188-5p inhibitors | CCCUCCACCAUGCAAGGGAUG |  |
| miR-188-5p inhibitors-NC | CAGUACUUUUGUGUAGUACAA |  |
| mir-188-5P mimics | 5’CAUCCCUUGCAUGGUGGAGGG 5’CUCCACCAUGCAAGGGAUGUU |  |
| mir-188-5P mimics-NC | UUCUCCGAACGUGUCACGUTT | ACGUGACACGUUCGGAGAATT |
| PTEN-siRNA | 5’GAGCGUGCAGAUAAUGACATT 5’UGUCAUUAUCUGCACGCUCTT |  |
| PTEN-siRNA-NC | UUCUCCGAACGUGUCACGUTT | ACGUGACACGUUCGGAGAATT |
| **Oligonucleotides used for Luciferase Assay** | | |
| PTEN-sense | AAACTAGCGGCCGCTAGTCAAATGAAGGGATATAAAAATAT | |
| PTEN-antisense | CTAGATATTTTTATATCCCTTCATTTGACTAGCGGCCGCTAGTT | |
| Mut-PTEN-sense | AAACTAGCGGCCGCTAGTCAAATGAATCTATATAAAAATAT | |
| Mut-PTEN-antisense | CTAGATATTTTTATATAGATTCATTTGACTAGCGGCCGCTAGTT | |

**Supplementary Table2:** List of Antibodies used in this study

| **Antibody** | **Cat #** | **Company** | **Dilution** |
| --- | --- | --- | --- |
| GAPDH | AB60004 | Proteintech | 1:1000 (WB) |
| αSMA | 55135-1-AP | Proteintech | 1:1000 (WB) 1:250 (IF) |
| COL1α2 | AB96723 | Abcam | 1:1000 (WB) 1:200 (IHC) |
| PCNA | 10205-2-AP | Proteintech | 1:1000 (WB) 1:200 (IF) 1:200 (IHC) |
| PTEN | 22034-1-AP | Proteintech | 1:1000 (WB) 1:300 (IHC) |
| AKT | 10176-2-AP | Proteintech | 1:1000 (WB) |
| pAKT | 66444-1-Ig | Proteintech | 1:1000 (WB) |
| BrdU | 66241-1-Ig | Proteintech | 1:300 (IF) |
| Anti-mouse Secondary Antiboy (WB) | 31430 | Pioneer | 1:5000 |
| Anti-Rabbit Sec. Ab. (WB) | SSA004 | Sinobiological | 1:5000 |
| Anti-mouse Sec. Ab. (IF) CoraLite 594 | SA00013 | Proteintech | 1:200 |
| Anti-Rabbit Sec. Ab (IF) Cy3 | EK022 | Zhuangzhi Bio | 1:200 |

*WB= Western blotting, *IF=Immunofluorescence, *IHC=Immunohistochemistry
